# Towards a Machine Vision-Based Yield Monitor for the Counting and Quality Mapping of Shallots

**DOI:** 10.3389/frobt.2021.627067

**Published:** 2021-04-16

**Authors:** Amanda A. Boatswain Jacques, Viacheslav I. Adamchuk, Jaesung Park, Guillaume Cloutier, James J. Clark, Connor Miller

**Affiliations:** ^1^ Precision Agriculture and Sensor Systems Laboratory (PASS), Department of Bioresource Engineering, McGill University, Sainte-Anne-de-Bellevue, QC, Canada; ^2^ Delfland, Inc., Napierville, QC, Canada; ^3^ Department of Electrical and Computer Engineering, McGill University, Montreal, QC, Canada

**Keywords:** precision agriculture, yield estimation, machine vision, watershed segmentation, shape detection, size estimation, quality assessement

## Abstract

In comparison to field crops such as cereals, cotton, hay and grain, specialty crops often require more resources, are usually more sensitive to sudden changes in growth conditions and are known to produce higher value products. Providing quality and quantity assessment of specialty crops during harvesting is crucial for securing higher returns and improving management practices. Technical advancements in computer and machine vision have improved the detection, quality assessment and yield estimation processes for various fruit crops, but similar methods capable of exporting a detailed yield map for vegetable crops have yet to be fully developed. A machine vision-based yield monitor was designed to perform size categorization and continuous counting of shallots *in-situ* during the harvesting process. Coupled with a software developed in Python, the system is composed of a video logger and a global navigation satellite system. Computer vision analysis is performed within the tractor while an RGB camera collects real-time video data of the crops under natural sunlight conditions. Vegetables are first segmented using Watershed segmentation, detected on the conveyor, and then classified by size. The system detected shallots in a subsample of the dataset with a precision of 76%. The software was also evaluated on its ability to classify the shallots into three size categories. The best performance was achieved in the large class (73%), followed by the small class (59%) and medium class (44%). Based on these results, the occasional occlusion of vegetables and inconsistent lighting conditions were the main factors that hindered performance. Although further enhancements are envisioned for the prototype system, its modular and novel design permits the mapping of a selection of other horticultural crops. Moreover, it has the potential to benefit many producers of small vegetable crops by providing them with useful harvest information in real-time.

## 1 Introduction

Crop yield estimation and mapping are important precision agriculture (PA) practices that can help growers efficiently keep track of their available resources and gain access to detailed representations of their farms. Accurate yield estimation allows growers to properly manage their harvest logistics, crop storage, sales, and account for losses in a timely manner (Nuske et al., 2014). Early and accurate predictions are also a key factor for market planning and trade (Bargoti and Underwood, 2017; Cheng et al., 2017). However, commercial PA techniques for specialty crops, such as fruits and vegetables, including yield monitoring systems are still uncommon. This is mainly due to a large diversity of harvesting methods for these types of crops. Furthermore, some of these crops are grown for smaller markets as compared to more prominent row crops like corn or soybean. Currently, yield estimation for specialty crops is often performed using manual sampling methods which are labor-intensive, time-consuming, and costly (Dorj et al., 2017; Nuske et al., 2014). Other methods rely heavily on imprecise historical or empirical data, which are then extrapolated either temporally or spatially (Cheng et al., 2017). Moreover, these calculations and measurements performed by humans are often prone to bias and sparsity leading to highly variable predictions (Bargoti and Underwood, 2017). Specialty crops are known to produce high value products, and the development of advanced yield monitoring systems would allow farmers to monitor crop quality better, to improve practices such as thinning using precise data, and to better estimate the size of the harvesting labor force, which would consequently lead to a reduction in operating costs (Patel et al., 2012).

Among the numerous sensing techniques used in PA, digital imaging is one that has been adopted in various applications such as robotic harvesting, weed control, phenotyping, pruning, seeding, spraying, thinning, sorting, and packaging (Kapach et al., 2012). Major applications of computer vision (CV) in agriculture have been developed for fruit detection, where the goal is to identify individual fruits, segment them from scenes with branches, foliage, or sky, and localize them in space for either yield estimation or for robotic harvesting systems (Kapach et al., 2012). A selection of studies have been performed in orchard environments using canopy images of fruits such as apples (Gongal et al., 2016; Linker et al., 2012; Wang et al., 2012; Zhou et al., 2012), oranges (Dorj et al., 2017; Hannan et al., 2009), mangoes (Payne et al., 2013), and berries (Nuske et al., 2014; Pothen and Nuske, 2016; Mirbod et al., 2016). For example, (Wang et al., 2012) created a stereo vision-based system using a two-camera stereo rig. This system was stationed on an autonomous orchard vehicle designed to work at night with artificial lighting. It converted apples to the Hue-Saturation-Value (HSV) color space, and then used features such as color and specular reflection to separate both red and green apples from foliage. The average errors obtained for crop yield estimation was −3.2% for red apple trees and 1.2% for green apple trees, respectively. (Gongal et al., 2016) developed an over-the-row machine vision (MV) system using both a Red-Green-Blue (RGB) and a stereo-vision camera which captured dual images from both sides of the plant canopy and localized apples in space. The experiment was performed in a controlled environment using artificial lighting and a tunnel structure covering the canopy. Using color segmentation, a Circle Hough Transform (CHT) and blob analysis, apples were identified in the images based on shape and color with an accuracy of 78.9%. More state-of-the-art methods (Bargoti and Underwood, 2017) have adapted machine learning techniques, such as Multi-Layered Perceptrons (MLPs) and Convolutional Neural Networks (CNNs), to perform pixel-wise fruit segmentation under natural sunlight in orchards using both an image segmentation based on a Watershed Transform (WST) and a CHT. The WST algorithm detected apples with a coefficient of determination (R^2^) of 0.83, and generated an apple yield map for an orchard block using an on-board NovAtel SPAN (NovAtel Inc., Calgary, Alberta, Canada) global positioning inertial navigation system (GPS/INS) which recorded the vehicle position and orientation with every image taken.

Although advanced CV systems have shown promising results, many external factors in farm image data can significantly hinder their performance. For example, farm image data is prone to large intra-class variations caused primarily by variable illumination conditions, occlusion by other crops or foliage, camera view-point, and seasonal maturity levels leading to crops with varying size, shape or color (Bargoti and Underwood, 2017; Hannan et al., 2009; Sengupta and Lee, 2014). Multiple detection of the same object within sequential images, or occlusion by other objects can lead to miscounting in yield calculation applications. (Gongal et al., 2016) used both a 2-dimensional (2D) and 3-dimensional (3D) imaging approach, where apple fruits were mapped together in a common coordinate system that correctly identified and removed duplicates. Fruits within the orchard were represented in a 3D space, where apples registered with the same X, Y, and Z coordinates were considered as one fruit. (Wang et al., 2012) developed a similar software to calculate the distance between every set of two apples and merged the apples as one whenever this distance was below a given threshold. (Hannan et al., 2009) used a centroid-based detection method to identify fruit clusters as a single fruit, and a perimeter-based detection method to locate the individual fruits with a success rate of 93% and a false detection rate of 4%. Another existing challenge is changes in object reflectance, which can make object detection unreliable and lead to incorrect or incomplete segmentation due to a non-uniform distribution of light intensity in the image. These types of problems can be addressed by creating a controlled, uniform lightning environment. Examples of such environments include an over-the-row platform with integrated LED lights (Gongal et al., 2016), a wooden box with a painted black interior (Al-Ohali, 2011) or simply performing the experiment at nightfall (Nuske et al., 2014; Wang et al., 2012). However, large farm experiments that require tractor operation are typically preferred during the day to not disrupt routine (Bargoti and Underwood, 2017), and are also more likely to be maintained over the long-term. It is therefore crucial that algorithms remain invariant to these factors to provide reliable outcomes leading to lasting practices.

Similar works aimed at computing the yield and quality assessment of vegetable crops in the field is currently scarce. One example includes work by (Kondo et al., 2009) who developed a MV system for the autonomous harvesting of tomato fruit clusters using stereo images of tomatoes in a greenhouse. The images were converted to the Hue-Saturation-Intensity (HSI) color space to generate chromacity distribution plots of H-versus-I. These plots were used to cluster fruit region properties and develop a classifier. The research results showed a 73% success rate in locating the stems of the clusters. Another study by (Blok et al., 2016) described an MV algorithm to identify broccoli heads on a farm. Texture and color-based segmentation were used to isolate the heads from the background. Results from the automatic segmentation method were compared with those obtained from two human experts by comparing the spatial overlap of the predicted and true broccoli head regions. The precision score of the segmentation was very high (99.5%) and overall accuracy of the image segmentation was 92.4%.

Spatial variability in soil type, soil fertility and other cropping conditions contribute to disparity in onion size, and onion size is an important limiting factor when determining the percentage of harvest destined to external suppliers. Many CV and MV methods have been proposed to non-destructively measure the size of various specialty crops (Al-Ohali, 2011; Gongal et al., 2016; Mirbod et al., 2016; Sengupta and Lee, 2014). Previous research has also made use of systems integrating different types of imagery such as thermal (Stajnko et al., 2004) or stereo-vision (Mirbod et al., 2016) to do this. Others employed time-of-flight-based 3D cameras (Gongal et al., 2016) to perform size determination and precision mapping of fruits in 3D space. Studies have been performed for quality inspection of sweet onions in a laboratory setting (Shahin et al., 2002; Wang and Li, 2014, Wang and Li, 2015), but work aimed at the automatic quality assessment and yield estimation of shallots has, to the present day, never been done. In this study, we investigate whether a machine-vision based approach could successfully classify onions by size during the harvesting process, and develop a novel and inexpensive system for this purpose.

## 2 Materials and Methods

### 2.1 Machine Vision System Design

#### 2.1.1 Data Collection

**FIGURE 1:**
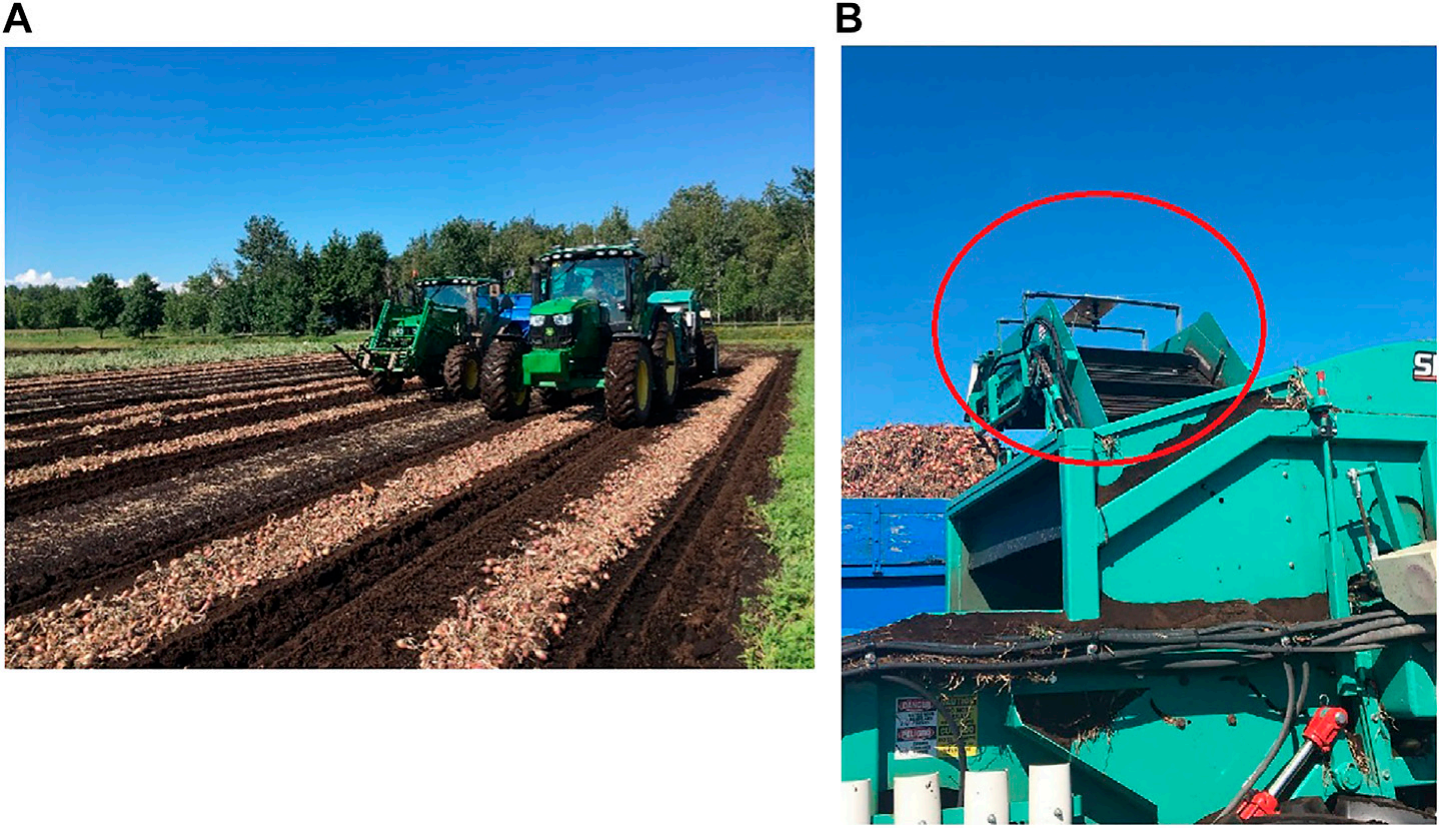
Shallot onion field **(A)** and machine vision bracket (encircled) **(B)**.

Shallot onions grow in clusters, where bulbs rest on the surface of the soil. They are harvested uniformly using a windrower and a trailer. A conveyor belt collects the onions from the ground using a paddle and the onions are then deposited in a trailer which stores them during harvest. This process is depicted in Figure 1A. The proposed system was mounted on a commercial shallot onion harvester (Univerco Inc., Napierville, Quebec, Canada) located on a shallot farm in Napierville, Quebec^1^ (latitude: −45.203603, longitude: −73.377636). Image data collection was performed on a sunny day with no clouds during the shallot harvesting season (September 24th) in the year 2018. A customized mounting bracket (Figure 1B) provided a vertical camera orientation, where the camera was facing downwards and directly above the conveyor at a 90◦angle. The bracket was positioned at the end point of the conveyor belt on the harvester to help reduce the amount of onions falling backwards and being detected more than once by the algorithm, while an on-board positioning system provided the geographic coordinates of every frame captured on the field (Figure 1B).

#### 2.1.2 Hardware Selection

**FIGURE 2:**
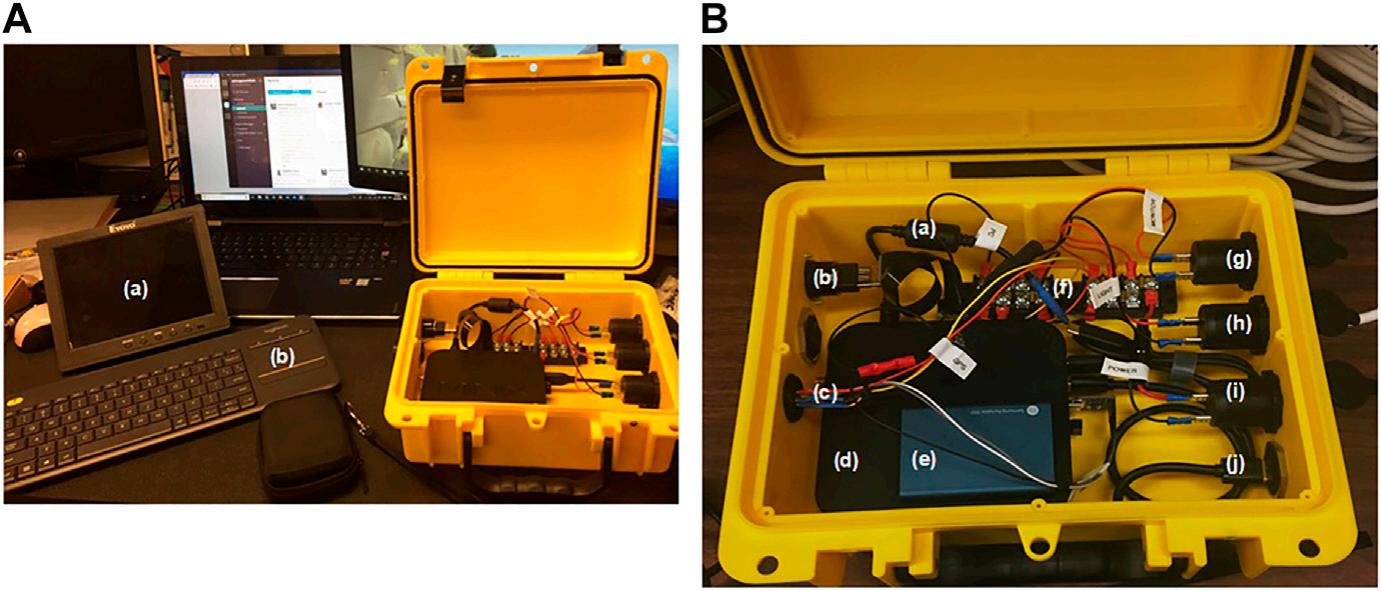
Yield Monitor. **(A)** shows the main components of the yield monitoring system including the monitor **(A)** and keyboard **(B)**. **(B)** depicts the components within the control box including the computer power line **(a)**, HDMI input **(b)**, GPS sensor attachment **(c)**, MINIX computer **(d)**, external solid-state drive **(e)**, terminal block **(f)**, 12-V power sockets **(g–i)**, and USB input for the webcam **(j)**.

For this project, size needed to be determined in terms of a standard 2D metric, and localization in space or even within the image was not necessary. Therefore, after careful analysis, an RGB complementary metal-oxide semiconductors (CMOS) high-resolution camera was chosen as the final sensor for this application to make image analysis less computationally expensive. An ELP 1080P USB Camera Box (Ailipu Technology Co., Ltd, Shenzhen, Guangdong, China) and a Garmin 19x HVS NMEA 0183 GPS sensor (Garmin Ltd., Olathe, Kansas, United States) were selected for image and location acquisition, and a MINIX NEO-Z83-4-PRO-VESA computer (MINIX, Kowloon Bay, Hong Kong) was used to perform image processing. Video data was recorded and processed in real-time at a resolution of 640 × 480 pixels and a frame speed of 120 frames/s. Frames were registered every 2–3 s and displayed on a small monitor (Figure 2A). Recording some of the images to disk allowed for the algorithm to be modified and adjusted even after the harvesting season was completed. A control box was designed to house all the electronics and computer hardware (Figure 2B) and protect the system from vibration and dust. The main structure of the box was a complete watertight and crush resistant Seahorse protective case (Seahorse, El Cajon, California, United States). The main processing unit was small enough to fit within the tractor cab while not inhibiting the driver, and overall cost of the system was very low ($ 1212 CAD) compared to the costs of traditional agricultural machinery. Full specifications of the camera and other hardware can be found in the Supplementary Material. A total of 1,180 images were collected during the trial (roughly 10 min of run time), and within this a subset of 246 (20% of the dataset) randomly selected images were used to evaluate the detection performance of the algorithm. Within this sample of images, 1,782 onions were manually identified.

#### 2.1.3 Watershed Segmentation and Shape Detection

**FIGURE 3:**
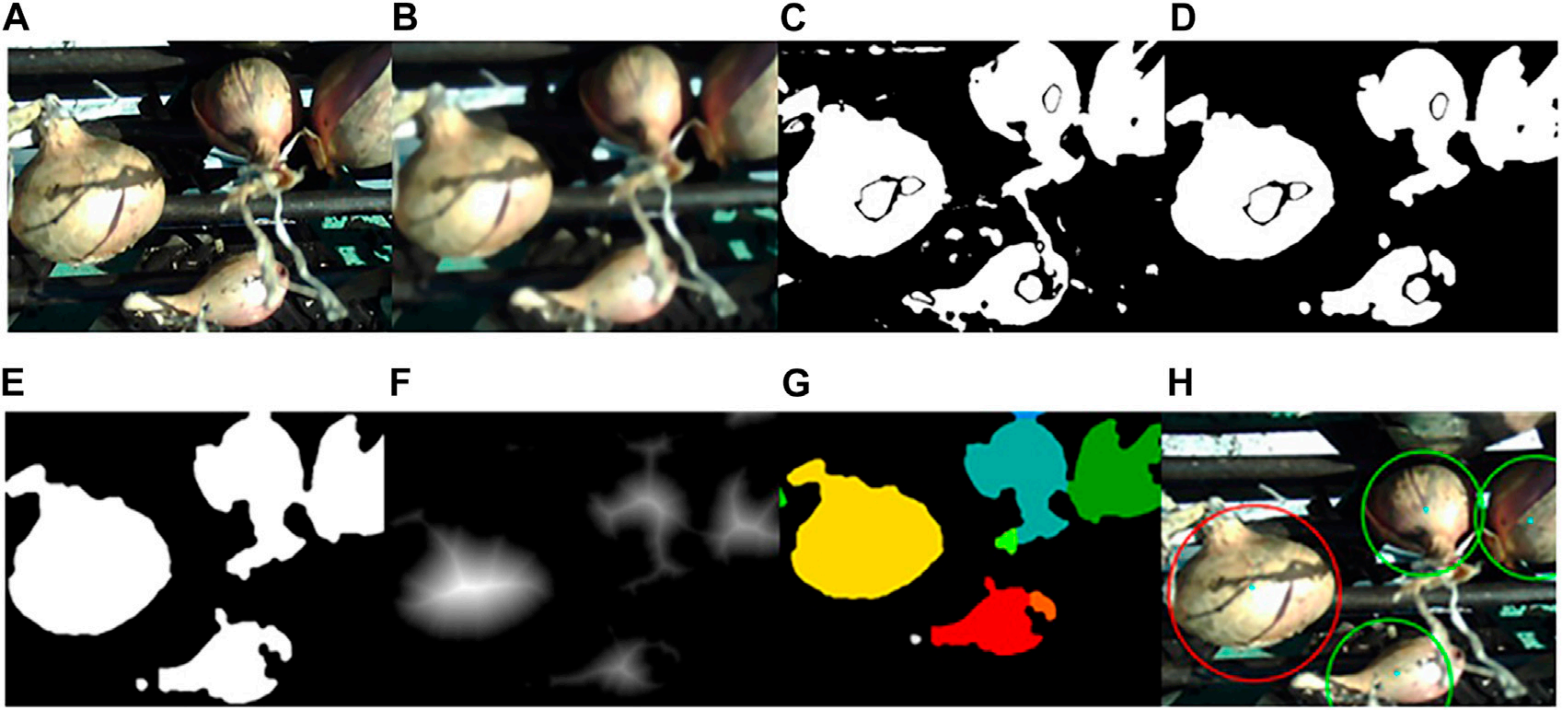
Segmentation results. The original image **(A)** is first preprocessed by blurring using a 9 x 9 median filter and 9 x 9 Gaussian filter **(B)**. It is then converted to the HSV color space and thresholded using a predetermined threshold value **(C)**. Morphological operations of opening **(D)** and closing are applied **(E)**, and the distance transform **(F)** is computed. Watershed segmentation is performed on the image to isolate individual onion regions **(G)** and identify and classify them in the original image **(H)**.

Figure 3 illustrates the pipeline used to identify onions and calculate their sizes. Preprocessing steps involved first blurring the original image (Figure 3A) with a 9 × 9 median filter to remove speckle noise, then blurring it once more with a 9 × 9 Gaussian filtering kernel. The default σ values in both the *x* and *y* axes were calculated using OpenCV’s cv2.getGaussianKernel() function for the selected kernel size (Figure 3B). Following this, the image was converted to the HSV color space and first segmented using color thresholding. Color regions of interest (ROIs) were determined by extracting the hue channel histogram from a sample of images, and identifying the pixel ranges corresponding to the onion skin [(0 ≤ H ≤ 40) and (160 ≤ H ≤ 180)] and white areas on the onion that exhibited specular reflection [(0 ≤ H ≤ 60) and (240 ≤ S ≤ 255)] (shown as blobs within the onions) (Figure 3C). This color segmentation approach was previously explored in (Boatswain Jacques et al., 2018).

Morphological operations of opening (Figure 3D) and closing (Figure 3E), both using a 12 × 12 elliptical structuring element, were applied to refine the onion regions and remove noise. Segmentation using a marker-based watershed transform (WST) was performed to improve separation of regions that were adjacent and overlapping (Meyer and Beucher, 1990; Vincent and Soille, 1991). In this segmentation approach, the image is interpreted as a topographical surface where the gradient image intensities are represented as elevations. Region edges are equivalent to watershed boundaries, and low-gradient region interiors are the catchment basins. The watershed segmentation algorithm attempts to group all pixels belonging to the same catchment basin, or region within the segmented image, using the distances of each binary pixel to the nearest 0-value (background) pixel, which is depicted using the distance transform (Figure 3F). Once these steps are completed, a marker-controlled watershed segmentation is performed by labelling the regions which are considered foreground with high confidence, or the “local peaks”, in the distance map (Sonka et al., 2015). Connected component analysis with 8-connectivity is performed on these peaks, and they are then passed to the watershed algorithm, which assigns a label to each region (Figure 3G) (Bishop, 2006). Finally, properties of these regions, such as size and contour length, were assessed before identifying them as onions and labelling them in the correct size category (represented by a given color contour) (Figure 3H). The full software for this project can be retrieved from our GitHub repository^2^.

### 2.2 Size Estimation

#### 2.2.1 Definition of Vegetable Size Categories

**FIGURE 4:**
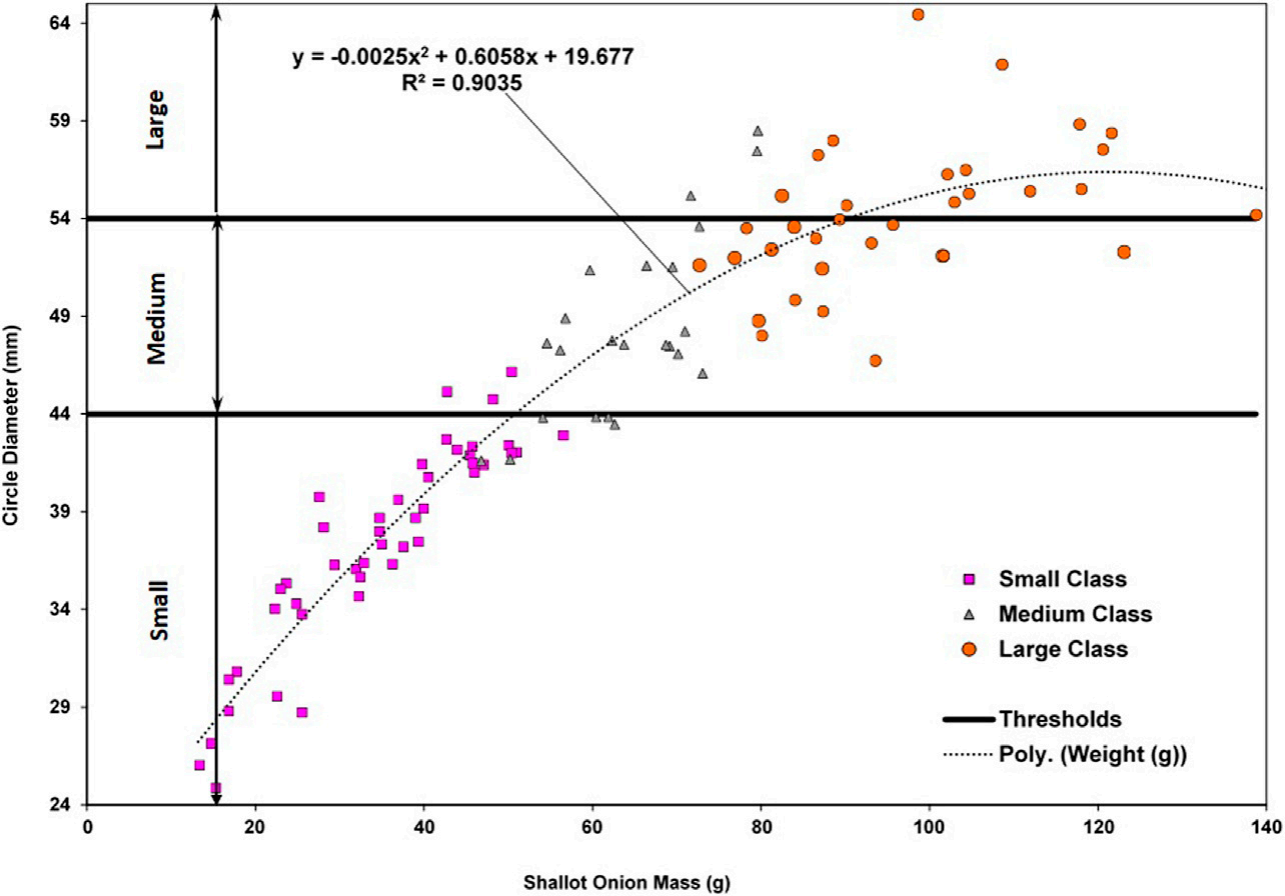
Determination of size classes for classification.

A sample of shallot onions (105) was collected from the field and analyzed by weighing each onion and measuring its diameter with a caliper. These onions were manually sorted by employees on the farm using visual observation, and were assigned a size class (small, medium or large) indicated by the markers in Figure 4. The size classes for the MV algorithm were then determined by plotting the diameter (mm) vs. onion weight (g). A high correlation was observed between onion diameter and onion weight (0.90), and therefore, diameter was utilized as the main characteristic to determine yield using MV. Size thresholds were then established to ensure that the two labels (visual observation and mathematically determined) coincided. Final thresholds determining the three size categories for the MV algorithm are also depicted as horizontal lines in Figure 4.

#### 2.2.2 Size Calibration

Once size classes were defined, a method was developed to automatically calculate the size of the onions in the images collected by the yield monitor. In this study, the requirement was to characterize onion size by generalizing values within three size categories. Therefore, a simple size calibration method was chosen. Real vegetable size can be estimated by calculating the area of the pixels occupied by the vegetable within the image and then directly correlating it to their real-world dimensions by using a reference object of known size (Al-Ohali, 2011; Stajnko et al., 2009). The reference object is isolated and measured in each image, and then a suitable pixel to metric ratio $P_{m}$ is calculated (Eq. 1). $P_{m}$ is determined as:

$$P_{m}=\frac{P_{d}}{T_{d}}$$ (1)

**FIGURE 5:**
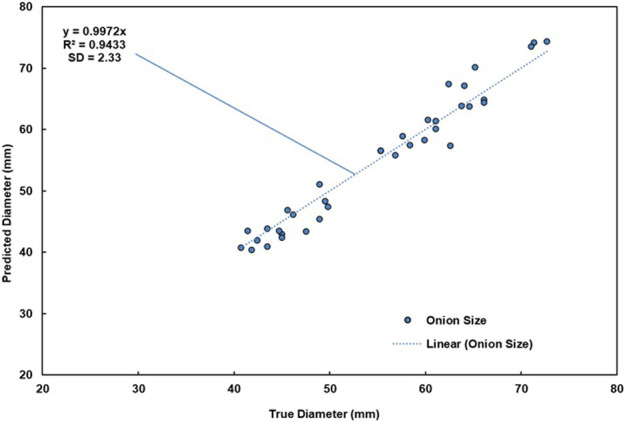
Size calibration results.

**FIGURE 6:**
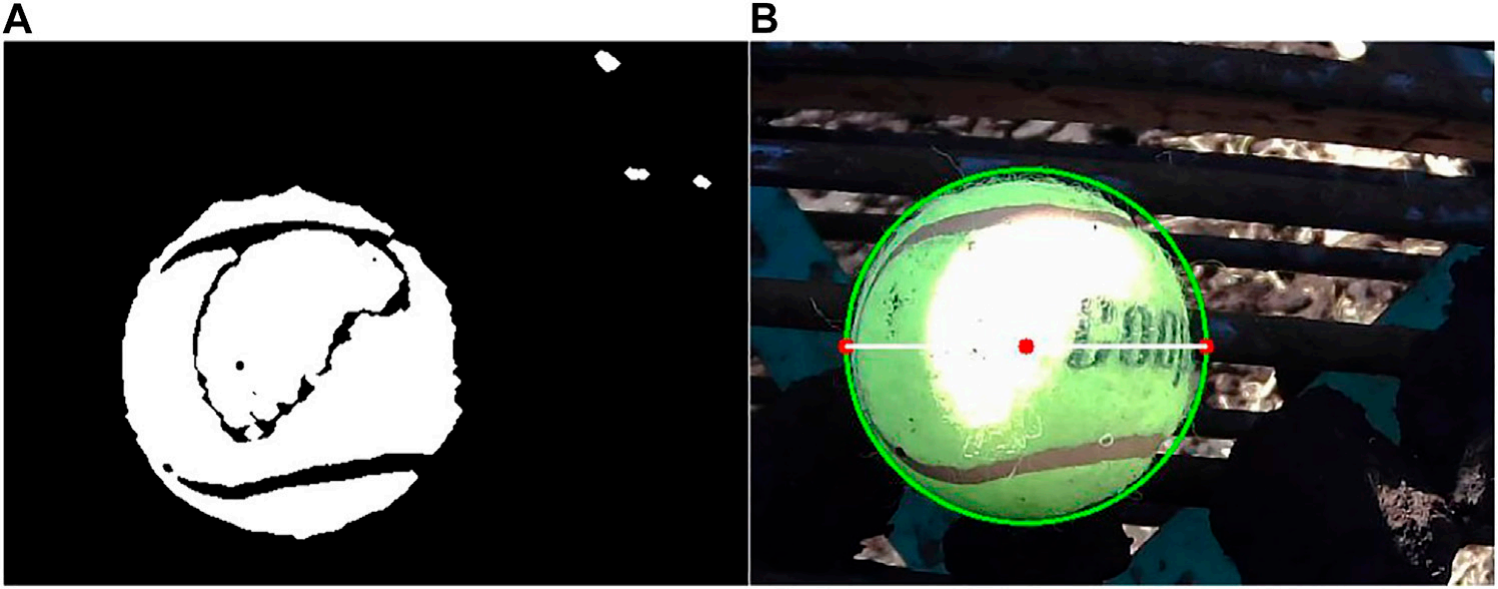
Size calibration segmentation results. Color thresholded result of the calibration object **(A)** and detection of the ball in the original image **(B)**. The diameter of the ball in pixel length is determined using a CHT.

Where $P_{m}$ is defined by taking the ratio of a pixel distance $P_{d}$ and the true value of this same distance $T_{d}$ in a real-life metric unit of choice. It is important that the dimensions of the reference object remain known, and that the object is easy to identify and segment from the image. A total of 35 onions were analyzed by comparing their true size, measured with a caliper, with the size predicted by the algorithm. A standard 300 mm ruler was used as the reference object for this test. Results showed a very high correlation (R2=0.94) between the predicted results and the true size values (standard deviation (SD) of 2.33 mm) (Figure 5). Therefore, this method was deemed suitable for the prototype during the in-field trials. A tennis ball was selected as the calibration object for the field trial due to its distinct yellow color which could easily be segmented even in the field. OpenCV approximates the ball using a CHT and extracts its diameter in pixel length. The recorded $P_{m}$ was roughly 3.38 pixels/mm. Figure 6A and Figure 6B show the results from this segmentation and the detection of the ball in a sample image from the conveyor, respectively.

Within the full dataset of images, a total of 92 images were randomly chosen to assess the performance of the size prediction method. Within these images, 271 (15.2% of all shallots present) shallots were analyzed by measuring the diameter of the automatically detected boundary of the bulb, and comparing this estimated value with the true pixel boundary size of the onion.

### 2.3 Algorithm Evaluation

#### 2.3.1 Performance Metrics

To evaluate the performance of the algorithm, a selection of performance metrics were used. *Accuracy* (Eq. 2) is an important metric that represents the capacity of an algorithm to correctly classify instances. Although it is a powerful indicator of overall performance, accuracy alone is not enough to determine the strength of an algorithm and whether it has correctly learned the task at hand (Baratloo et al., 2015). Accuracy is defined as:

$$Accuracy=\frac{\Sigma TP+\Sigma TN}{\Sigma TP+\Sigma TN+\Sigma FP+\Sigma FN}$$ (2)

where TP and TN are the number of true positive and true negative classifications, respectively; and FP and FN are the number of false positive and false negative classifications, respectively. Accuracy, like all metrics, is often multiplied by 100 to yield a percentage. The observed error was modelled by calculating the TP, FP, TN, and FN counts from the visual observations. TPs corresponded to onions that were correctly detected, FPs were other objects (background, stems, rocks) that were falsely classified as onions. Finally, FNs were onions that were missed by the detection algorithm.

*Precision* (Eq. 3), also referred to as positive predictive value (PPV), is used to determine the capacity of an algorithm to correctly identify positive cases with respect to all the cases the algorithm has classified as positive. It is calculated by dividing the number of true positives by the number of predicted positives, which itself is a sum of TP and FP (Baratloo et al., 2015). Precision is defined as:

$$Precision=\frac{\Sigma TP}{\Sigma TP+\Sigma FP}$$ (3)

Precision is an indicator of how reproducible and repeatable a measurement is under unchanged conditions and is used to evaluate the exactness of a model.

*Recall* (Eq. 4) is the fraction of relevant instances that have been correctly identified (TP) over the total amount of relevant instances (TP and FN). Recall and precision are typically used in unison to report the performance of a classification system. Precision indicates the quality of the positive prediction capability of the model, while recall indicates the completeness or quantity of correct predictions with respect to all positive instances present. High precision would mean that the algorithm returned a greater amount of relevant results than irrelevant ones, while a high recall value would mean that the algorithm returned most of the relevant results (Buckland and Gey, 1994; Baratloo et al., 2015).

$$Recall=\frac{\Sigma TP}{\Sigma TP+\Sigma FN}$$ (4)

*Standard Deviation*, also known as SD or *σ*, represents the dispersion of a dataset (or variability) with respect to its mean, and indicates how accurately a given mean value represents the sample data. The formula for SD is defined by Eq. 5:

$$SD=\sqrt{\frac{{\sum_{i=1}^{n}\left(\hat{x_{i}}-\bar{x}\right)}^{2}}{n-1}}$$ (5)

Where *n* is the number of observations, $\hat{x_{i}}$ is the i^th^ observation, and $\bar{x}$ is the sample mean.

*Root Mean Square Error* (RMSE) is the standard deviation of the residuals or prediction errors. RMSE is used to measure the difference between values predicted by a model and the values observed.

$$RMSE=\sqrt{\frac{{\sum^{\text{​}}}_{i=1}^{n}{\left(\hat{y_{i}}-y_{i}\right)}^{2}}{n}}$$ (6)

where *n* is the number of observations, $\hat{y_{i}}$ is the predicted value of the i^th^ observation, and $y_{i}$ is the observed value.

Lastly, *Standard Error of the Mean* (SEM) is calculated as the standard deviation of the distribution associated with that error, or an estimate of that same standard deviation (Eq. 7).

$$SEM=\frac{s}{\sqrt{n}}$$ (7)

where *s* is the sample SD and *n* is the sample size. SEM is used to approximate the uncertainty around the estimate of the mean measurement, and it is most useful as a means of calculating a confidence interval (Altman and Bland, 2005).

#### 2.3.2 Statistical Analysis

A two sample *Kolmogorov-Smirnov* (KS) test was applied to determine whether the predicted diameter values for each size category would differ significantly from the manually measured values (Dodge, 2008). The KS test statistic is defined as the maximum distance *D* between the cumulative distribution curves of a set of samples, and is given by Eq. 8:

$$D=\text{max}\left(F\left(Y_{i}\right)-\frac{i-1}{n},\frac{i}{n}-F\left(Y_{i}\right)\right)\;\text{with}\;1\leq i\leq n$$ (8)

where *F* is the theoretical cumulative distribution being tested and $Y_{i}$ is a given observation within the total number of observations *n*. To use the KS-test, the distribution must be continuous and fully specified. A two-tailed test was performed with the following conditions:

H0: The two samples are drawn from the same distribution.
H1: The two samples are not drawn from the same distribution.

The KS statistic was computed using the two-sided asymptotic KS distribution.

## 3 Results and Discussion

### 3.1 Onion Detection Results

The WST method automatically detected 1,467 vegetables, with a total of 1,115 of these detections corresponding to true onions. This lead to an overall precision of 76.0%. Table 1 summarizes the performance of the WST detection algorithm. From these results, we can see that precision and recall of the selected algorithm reached acceptable rates, meaning that the algorithm correctly detected onions with reasonable confidence. The SEM for correctly detected onions was 0.149 for a total number of 246 observations. On average, a total of 6.85 onions were present within an image, and the algorithm could properly detect 4.29 (62.6%). However, overall accuracy evaluated on the entire test set was 59.2%.

**TABLE 1:** Summary of detection performance metrics.

Metric	Score
Precision	0.760
Recall	0.727
Overall Accuracy	0.592

**FIGURE 7:**
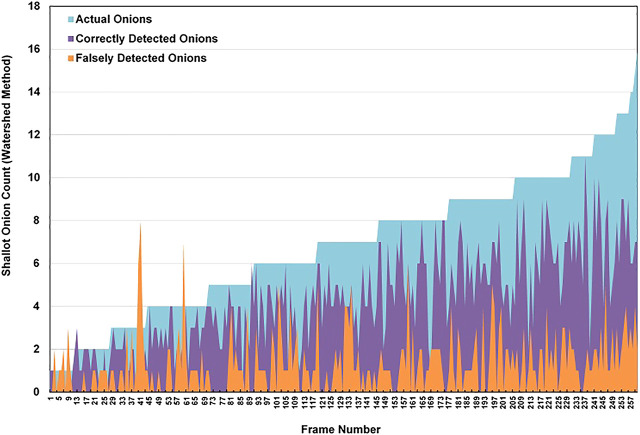
Detection results for the WST method.

Results of the error analysis are featured in Table 2. Figure 7 also demonstrates the behavior of this error with respect to the total number of actual onions, correctly detected onions and falsely detected onions in a given frame. As the onion count in the image remained low, the predictions remained accurate. However, as the count per image gradually increased beyond nine onions, the algorithm began to miss more vegetables. Possibly, this could be caused by the improper segmentation of vegetables that occurs when the image is cluttered due to a large portion of onions overlapping.

**TABLE 2:** Summary of Type I and Type II error distribution for the Watershed Method.

	Type I error (falsely detected onions)	Type II error (missed onions)
Total Sum	352	418
Mean (per frame)	1.35	1.61
Median	1.00	1.00
Standard Deviation	1.41	1.81
Max	8.00	8.00
Min	0.00	0.00

**FIGURE 8:**
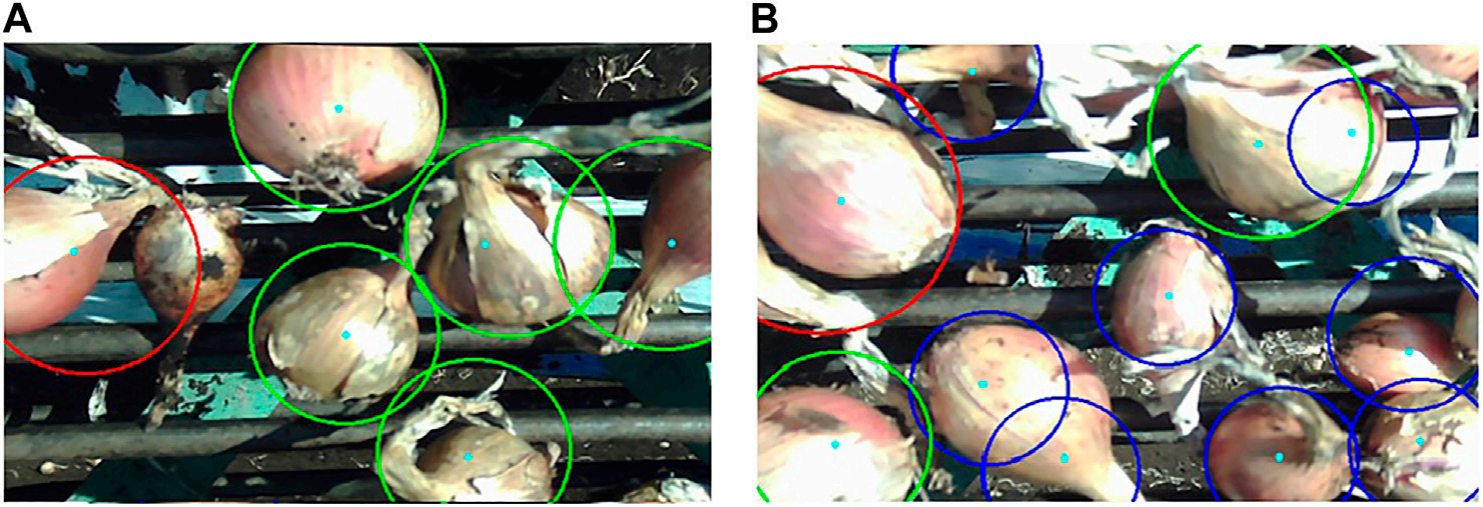
Two example frames showing onion detection results. Size categories are indicated by colored circle boundaries (Blue = small, Green = medium, and Red = large).

The WST segmentation method failed mostly when there were large reflective areas present on the onion, which would appear almost entirely white and hence, they were not properly captured by color thresholding. Bright spots in some images were caused by inconsistent lighting, which also led to false detections. In some extreme cases, the original image was overly saturated with light making the onion regions appear almost fully white. This would leave large holes within the thresholded image of the onion which were not filled even after noise removal with opening/closing, preventing the allocation of a single minimum to that specific region. The algorithm also missed some onions that were partially visible and on the border of the image, onions occluded by larger bulbs or stems, or bulbs that were present in shadowy regions. False detections corresponded to onions clustered in the trailer that were visible through the openings of the conveyor paddles, or onions that were improperly segmented causing the same bulb to be identified twice. Some of these cases can be observed in Figures 8A,B.

### 3.2 Size Estimation Results

Tables 3, 4, 5 show a full description of the predicted (CV determined) vs. true diameter distributions for each size category. The best size classification performance was within the large class (73.3%). However, this class was significantly less predominant than the other classes with a small number of samples (30). Large shallots are typically less common, since most producers have determined that consumers prefer the small to medium sized vegetables. The size prediction accuracy of the small class, with 88 correct predictions out of 150 small onions, was equal to 58.6%. The least correct classification rate was found within the medium onion category (44.4%). The mean difference between the predicted and true values of the medium class (1.68 mm) was lower in absolute value than that of both the small (6.86 mm) and large classes (2.06 mm). Nevertheless, results showed that the overall performance of this algorithm was poor (R2 = 0.011, results not shown) with 55.9% of onions correctly classified and a Root Mean Square Error (RMSE) of 11.3 mm.

**TABLE 3:** Kolmogorov-Smirnov test results for the small class.

	Diameter (predicted)	Diameter (true)
Mean (mm) (*N* = 150)	43.87	37.00
Median (mm)	42.50	37.32
Min (mm)	29.32	23.40
Max (mm)	76.12	43.83
Standard Deviation (mm)	10.41	1.62
Mean Difference (mm)	6.86	
KS Test Statistic	0.4199	
*p*-Value	2.849E-12	

**TABLE 4:** Kolmogorov-Smirnov test results for the medium class.

	Diameter (predicted)	Diameter (true)
Mean (mm) (*N*=90)	49.46	47.78
Median (mm)	49.76	47.39
Min (mm)	29.91	44.13
Max (mm)	77.30	53.61
Standard Deviation (mm)	10.44	2.75
Mean Difference (mm)	1.68	
KS Test Statistic	0.3222	
*p*-Value	1.191E-4	

**TABLE 5:** Kolmogorov-Smirnov test results for the large class.

	Diameter (predicted)	Diameter (true)
Mean (mm) (*N*=30)	58.44	60.51
Median (mm)	58.20	57.01
Min (mm)	39.09	54.20
Max (mm)	74.93	87.67
Standard Deviation (mm)	8.12	8.79
Mean Difference (mm)	−2.06	
KS Test Statistic	0.2333	
*p*-Value	0.3420	

From Figure 4, we can see that there is inherent bias in the manually determined size categories. This is especially noticeable around the border area between the medium and large classes, where there is more variation in onion size for a given weight. For the algorithm to work properly, it would have to correctly separate the three size classes, while leaving as little overlap as possible between them. More onion samples will need to be taken and measured to create a better representation of the vegetable size distribution and redefine these manual thresholds. These results further support the idea of perhaps considering a non-linear method to separate the size classes. This could be done by modelling the vegetables as ellipses instead of circles and sorting the onions by the major-axis and minor-axis values in a 2D setting, and not solely by diameter. However, this would slightly increase computation time.

Following are the results of the two-sample KS tests. The KS statistic was computed using the two-sided asymptotic KS distribution. If the *p*-value was shown to be above the KS test statistic within a given threshold, then the null hypothesis (stated in *Statistical Analysis*) could not be rejected. Results from the statistical analysis for each size class are also tabulated (Tables 3, 4, 5). According to these results, the null hypothesis is not rejected for the large class with a confidence interval of α = 0.05. Therefore, the algorithm can correctly predict large onions at an acceptable rate. This may be due to the increase in overlap that occurs when there are many small onions in a frame. However, the null hypothesis is rejected for both the small and medium classes with a confidence interval of α = 0.05.

The present results are significant in at least two major respects. The first is that here we present a relatively simple, computationally inexpensive and low-cost device. At the time that the system was built, most MV methods explored to develop the algorithm used more deterministic approaches based on feature engineering which can explain some of the limitations that can be witnessed. However, its potential is apparent given that, presently, there are no existing commercially available alternatives to do quality mapping of shallots. Although, accuracy is lacking in a set of situations explained in the previous sections and this will have to be corrected. Secondly, this system lays down the foundations needed to produce an even more interesting and robust device that could be used in a broad range of settings. It demonstrates the possibility and feasibility of such a system, and opens the door to the multiple perspectives that could lead to significant improvement.

## 4 Future Improvements

A disadvantage of the current system relates to the effectiveness of the size calibration process. Once the yield monitoring program was started, calibration images had to be taken by placing a tennis ball in front of the camera and recording a small set of frames that would later be used to establish a pixel metric. However, the thresholds for the color segmentation of the tennis ball were set with images from a previous run. Therefore, the segmentation could sometimes be faulty depending on the existing lighting conditions of the consecutive runs. A threshold setting method capable of being adjusted in the field would improve color thresholding based on the current lighting conditions. Another option might be to use the distance between paddles to calibrate the size estimation algorithm, although this would require the development of a new method to segment the paddles from the image.

**FIGURE 9:**
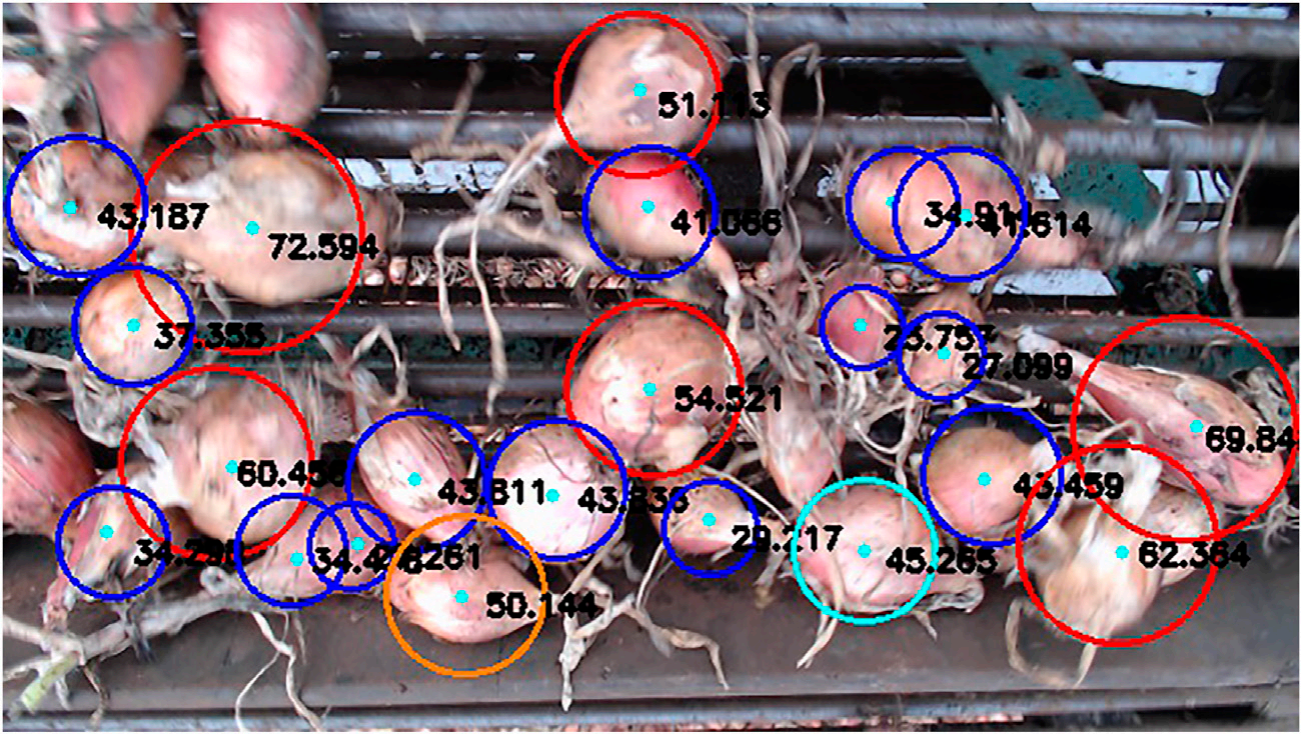
Examples of onion detection results using *k*-means clustering of pixels in the HSV color space with *k* = 6. Predicted onion sizes (mm) are marked directly on the image.

Further enhancements of the algorithm must be made to improve the segmentation of individual onion regions and to increase overall accuracy. One way to enhance this could be the development of a more resilient algorithm using a form of machine learning approach based on fully-connected CNNs called semantic segmentation. A structure like that of (Bargoti and Underwood, 2017) could potentially learn features that could accommodate for all variability in the appearance of the onions discussed in *Onion detection Results*. However, this would require the labelling of possibly thousands of images, which can prove to, in itself, be costly. Other alternatives could be to use unsupervised learning techniques such *K*-Means clustering or Decision Trees to perform segmentation. Some work has been done in this area and has shown to be promising (Figure 9). However, it is not at a stage where it could be fully presented within this article.

The system did occasionally run into power problems and had to be rebooted to resume data collection. This was mainly due to some power connections being disconnected while the tractor was in motion. Hardware adjustments are needed to develop a more reliable system that can withstand the harsh conditions of the agricultural environment. These would include more stable power connections and a weatherproof camera capable of withstanding heavy vibrations, dust, moisture and variable lighting.

Enhancements to the algorithm are needed to improve overall detection rates while maintaining a low number of false positives. This may be done analyzing sequential frames and monitoring them for onions that appear in multiple frames using a visual-tracking algorithm such as a Kalman Filter for example.

## 5 Conclusion

Providing quality and quantity assessment of shallot onion crops during harvesting is crucial for securing higher returns and establishing more efficient management practices. This research focused on the use of computer vision as an alternative for yield estimation practices for specialized vegetable crops. A fully functional system was developed to record image and position data of shallot onion bulbs during harvesting and to create a geo-tagged image database for precision yield mapping. A computer software was developed to detect shallots in images and determine their sizes. The system used a watershed segmentation method and had a precision of 76% and recall of 73% on a sample of images. The software also reliably categorized large sized shallots with an accuracy of 73.3% but was limited when predicting small (58.6%) and medium (44.4%) onion sizes. This was primarily due to the improper boundary definition of bulbs that were either on the border of the image, overlapping or occluded by other bulbs or stems, or located in shadowy regions.

The incorporation of computer vision into agriculture is growing. Although further development is envisioned for this current system, it will help producers manage their harvesting strategies more efficiently. It served as a low-cost initial prototype which provided insight regarding the feasibility and economic potential of such systems. More care will be taken to produce a second prototype which could increase the reliability of the system and deliver a better product that could be used over the long-term.

## Data Availability

The original contributions presented in the study are included in the article/Supplementary Material, further inquiries can be directed to the corresponding authors.

## Footnotes

1
https://www.delfland.ca/en/

2
https://github.com/AmandaBoatswain/cv-based-yield-monitor
