# Supplementary material for: Towards a Machine Vision-Based Yield Monitor for the Counting and Quality Mapping of Shallots
Source: Front Robot AI. 2021 Apr 16;8:627067. doi: 10.3389/frobt.2021.627067 (PMC8146908; doi:10.3389/frobt.2021.627067)

Development of a Machine Vision Yield Monitor for Shallot Onion Harvesters

Amanda Boatswain Jacques, Viacheslav Adamchuk, Guillaume Cloutier, James J. Clark and Connor Miller

14th International Conference on Precision Agriculture
June 26th 2018
Montreal, Quebec, Canada

Introduction – Crop Yield

- Also known as agricultural output
- Measure of the production of a crop per unit area of land
- Some important factors influencing crop yield include:

  - Soil fertility
  - Pesticides
  - Fertilizers and nutrients
  - Weather

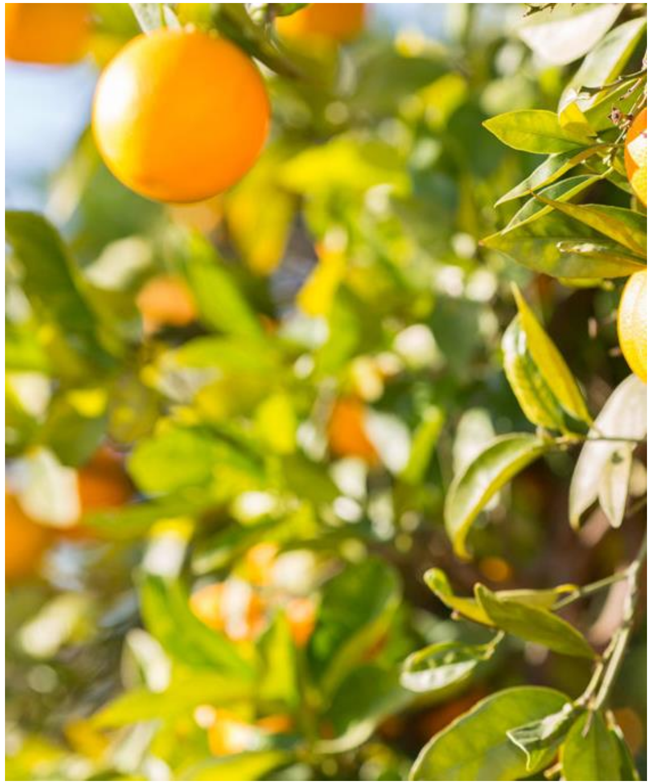

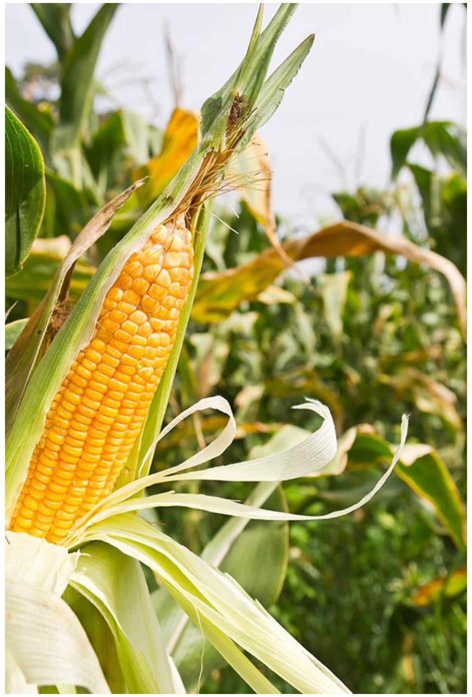

Why is knowing crop yield important?

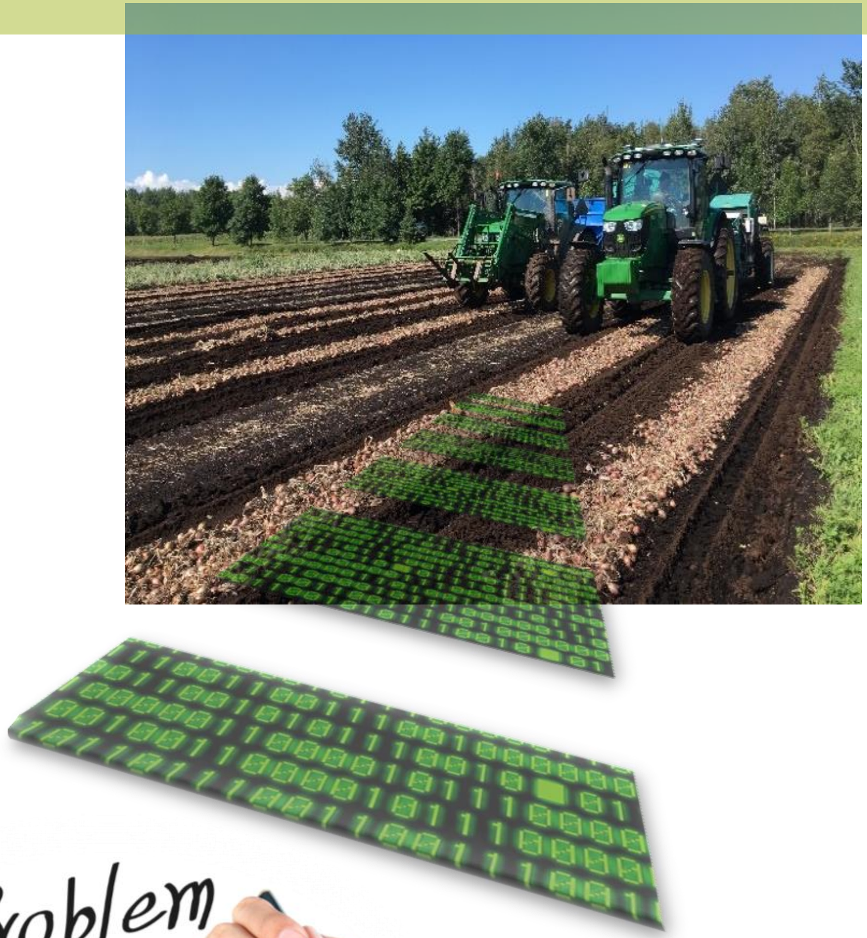

Crop yield estimation and mapping can help shallot onion growers:

- Spatial variabilities other cropping conditions result in great variability in onion size
- Quality assessment of shallots is done by human visual inspection
- Manage harvest logistics and crop storage
- Manage crop sales and account for losses sooner
- Key factor for market planning

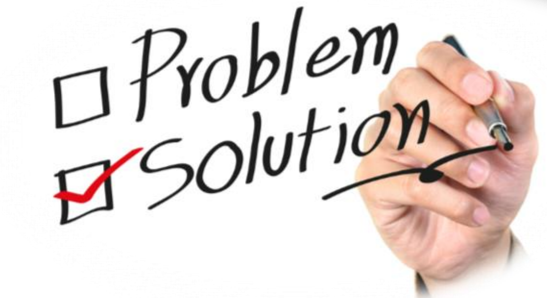

Methodology

- A typical MV system is composed of:
  I. An image processing module
  II. A pattern recognition module (blobs, edges, corners or lines)
- External factors can heavily influence results
- Some solutions include creating a controlled lighting environment, a platform with integrated LEDs or performing the experiment at night

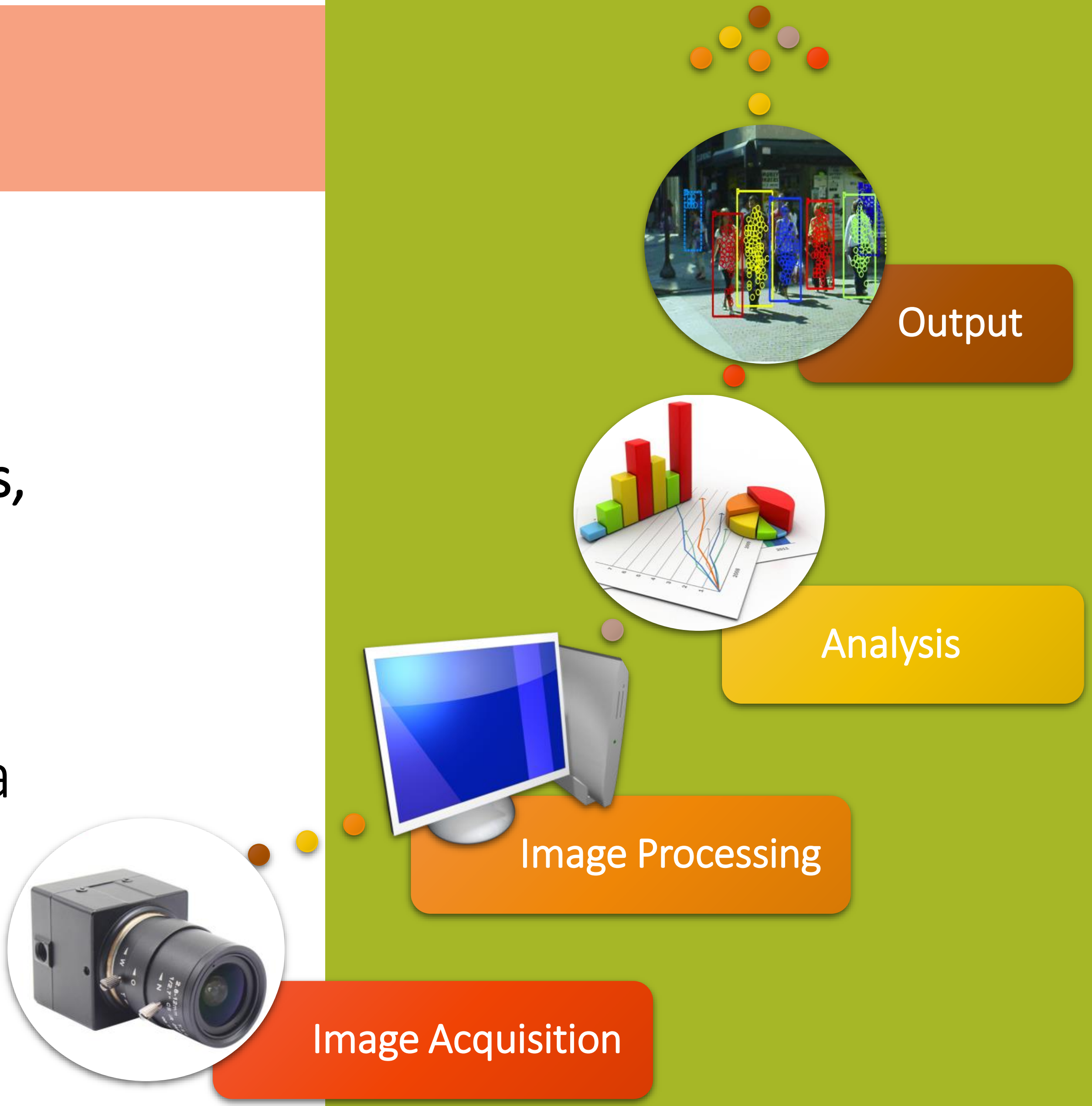

Methodology – System Design

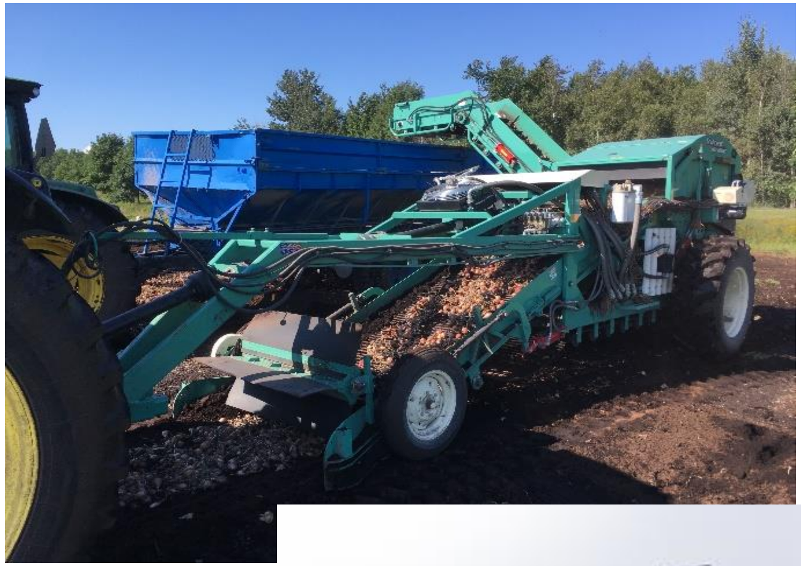

A customized bracket provides a vertical camera orientation, capturing an image where the camera is facing downwards and directly on the conveyor

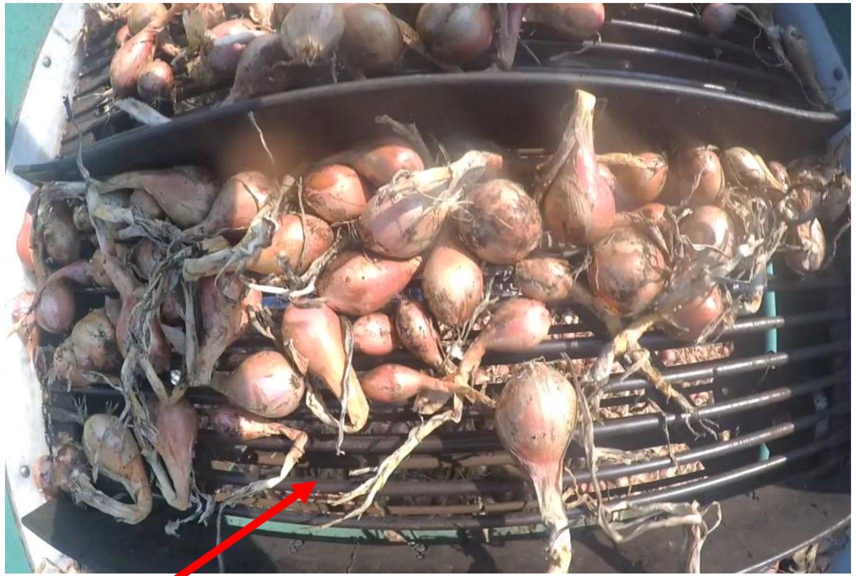

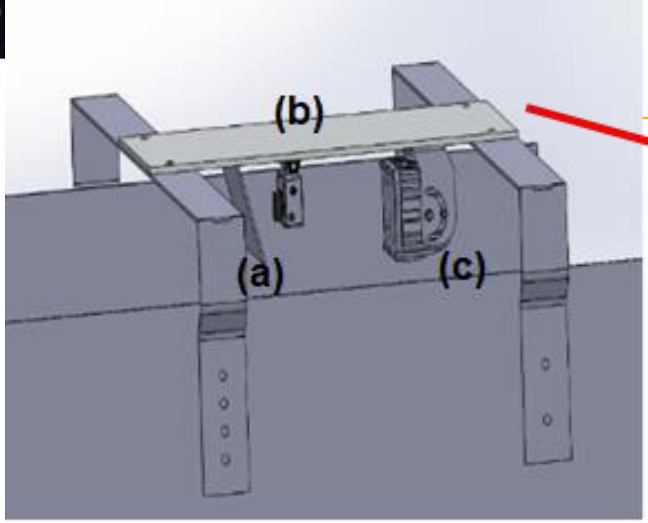

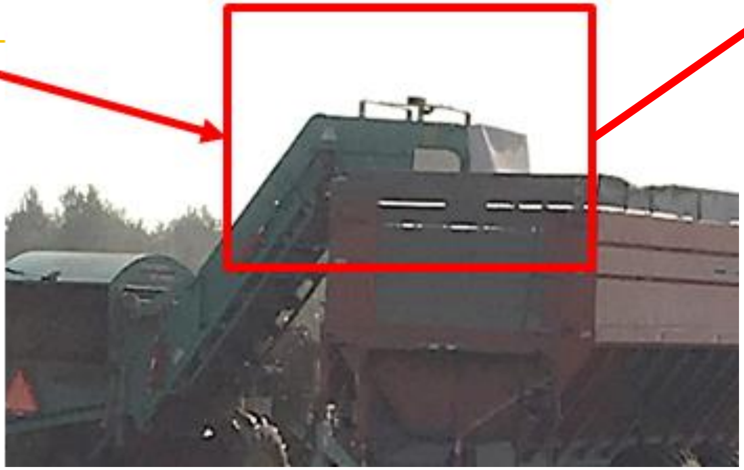

Methodology - Algorithm

Data Acquisition → Image Preprocessing → Segmentation → Noise Filtering

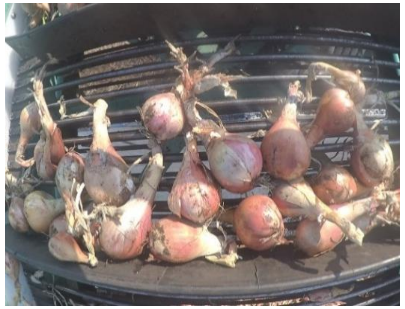

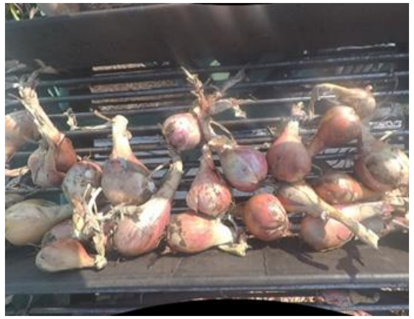

Results - Segmentation

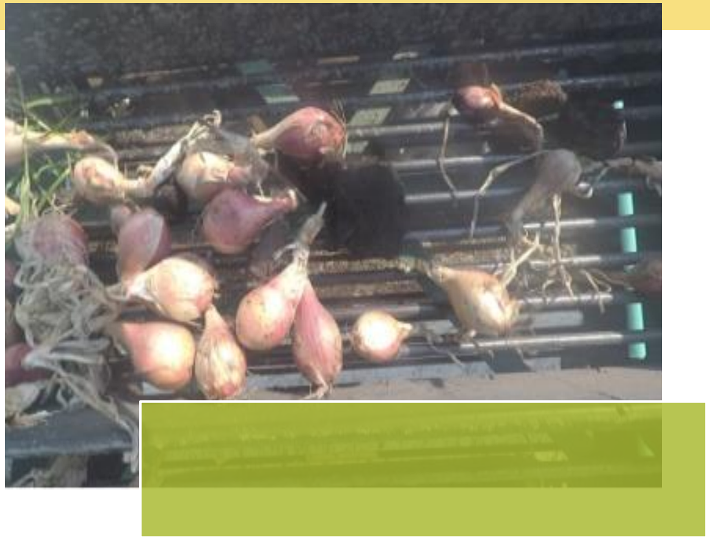

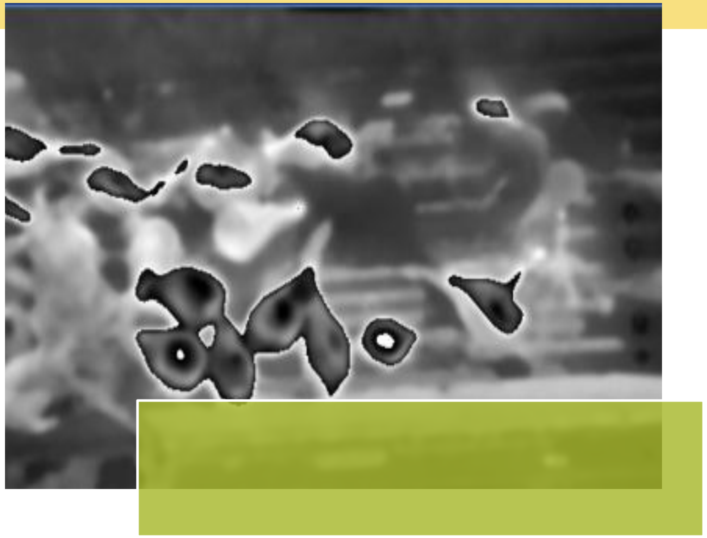

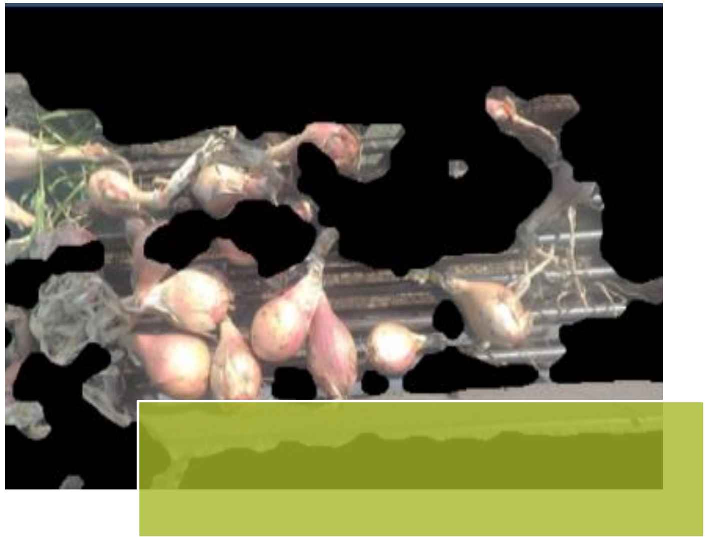

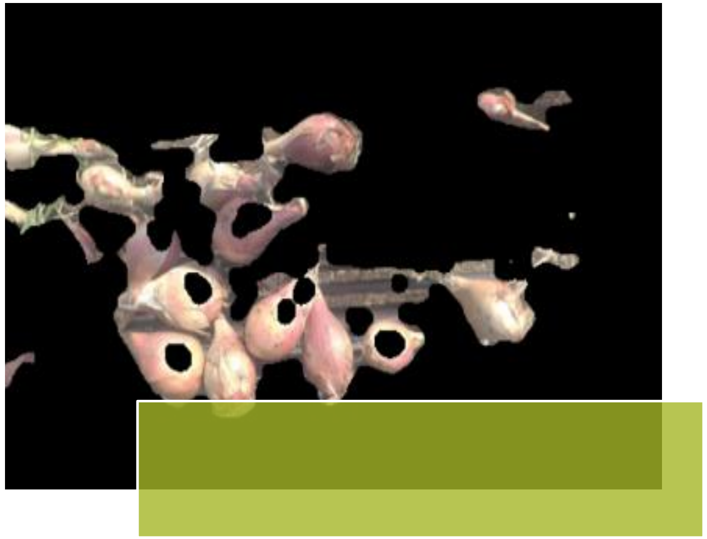

Results

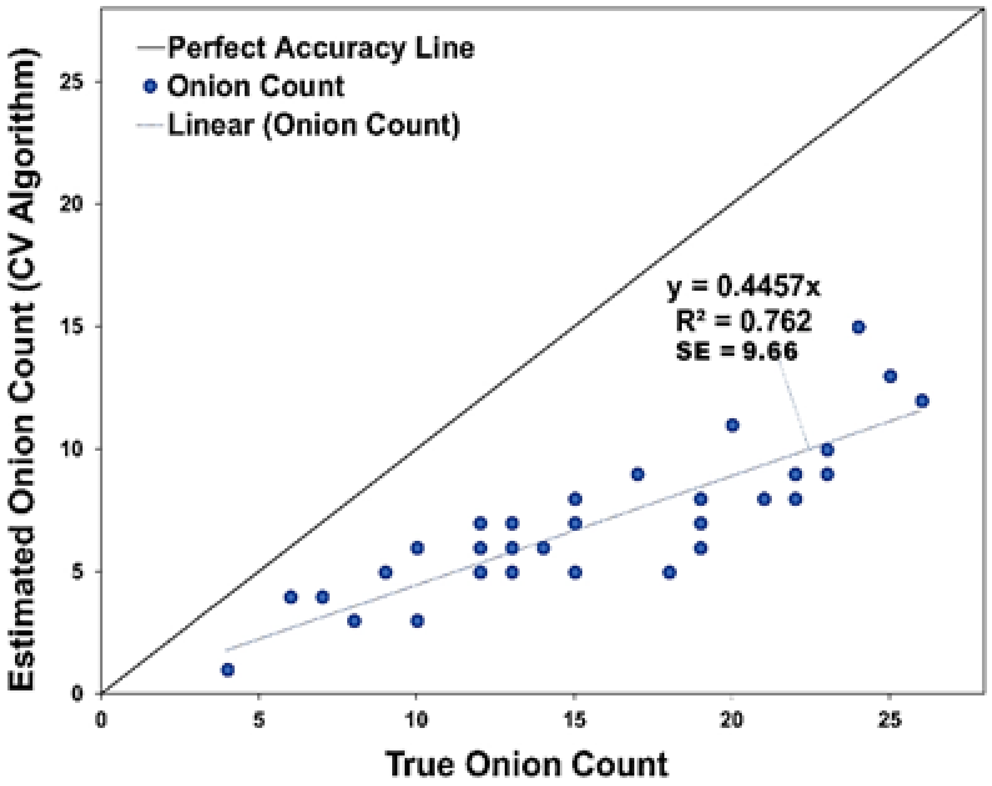
Perfect Accuracy Line
Onion Count
Linear (Onion Count)
y = 0.4457x
R² = 0.762
SE = 9.66
Estimated Onion Count (CV Algorithm)
True Onion Count
0
5
10
15
20
25

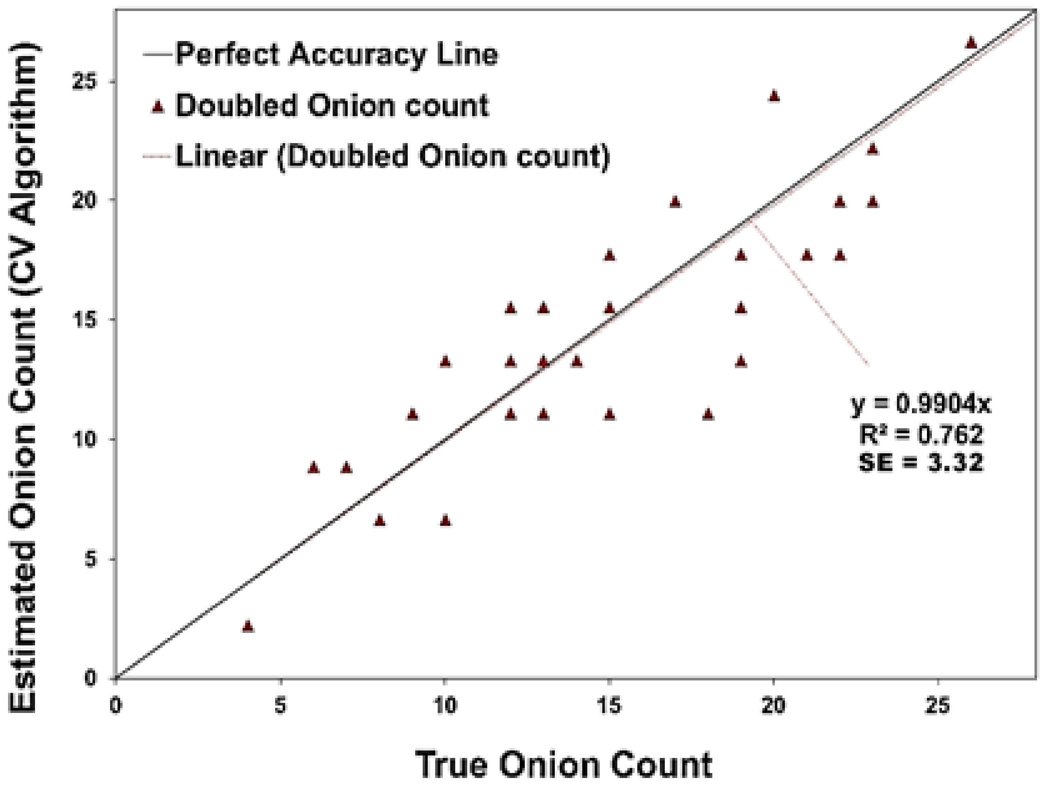
Perfect Accuracy Line
Doubled Onion count
Linear (Doubled Onion count)
y = 0.9904x
R² = 0.762
SE = 3.32
Estimated Onion Count (CV Algorithm)
True Onion Count
0
5
10
15
20
25

Results

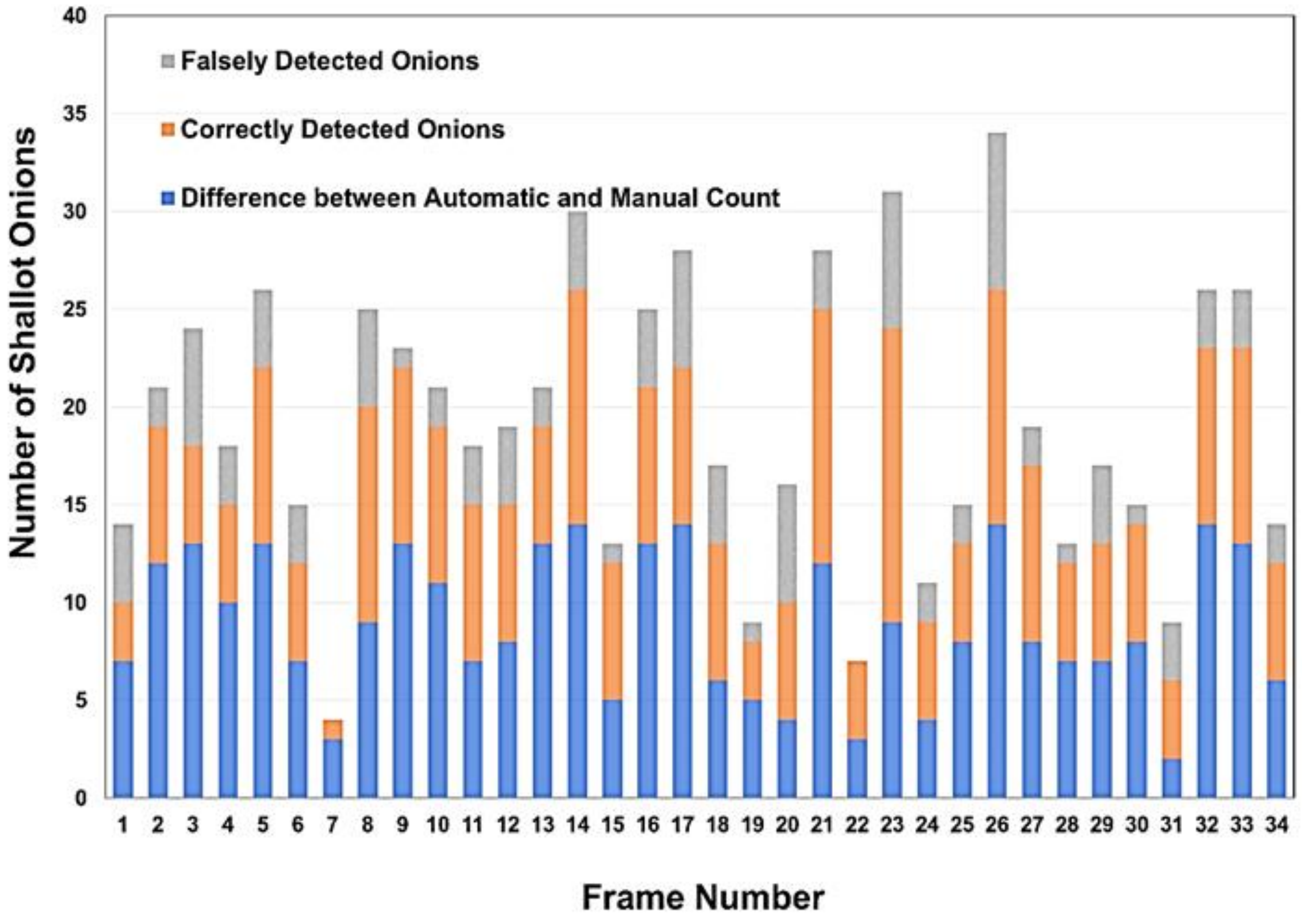

Falsely Detected Onions
Correctly Detected Onions
Difference between Automatic and Manual Count
Number of Shallot Onions
Frame Number
0
5
10
15
20
25
30
35
40
1 2 3 4 5 6 7 8 9 10 11 12 13 14 15 16 17 18 19 20 21 22 23 24 25 26 27 28 29 30 31 32 33 34

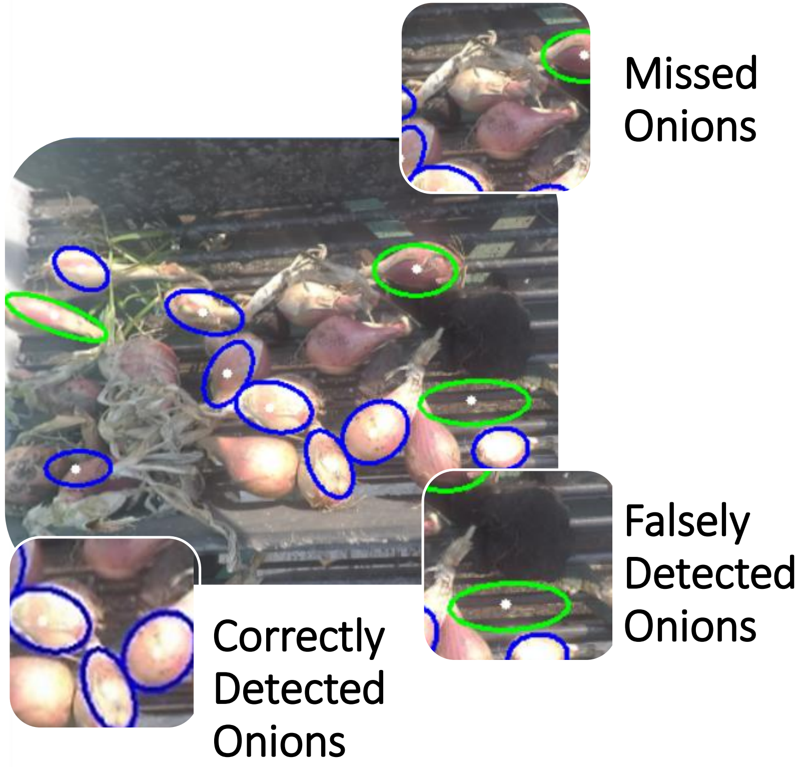

Missed Onions
Falsely Detected Onions
Correctly Detected Onions

Conclusion

Although further development is envisioned for this system, it will help producers manage their harvesting strategies more efficiently and increase their revenue

Producers could also gain access to important data relating the performance of their field and improve their ability to detect irregularities more frequently and easily

Continuous progress made in pattern recognition and image processing methods permits many MV systems to be built for grading and sorting almost any agricultural product, including the vegetables considered during the progression of this study

Acknowledgements

This research was supported in part by the company Defland, Inc. and by the Mitacs Accelerate internship program.

Questions?

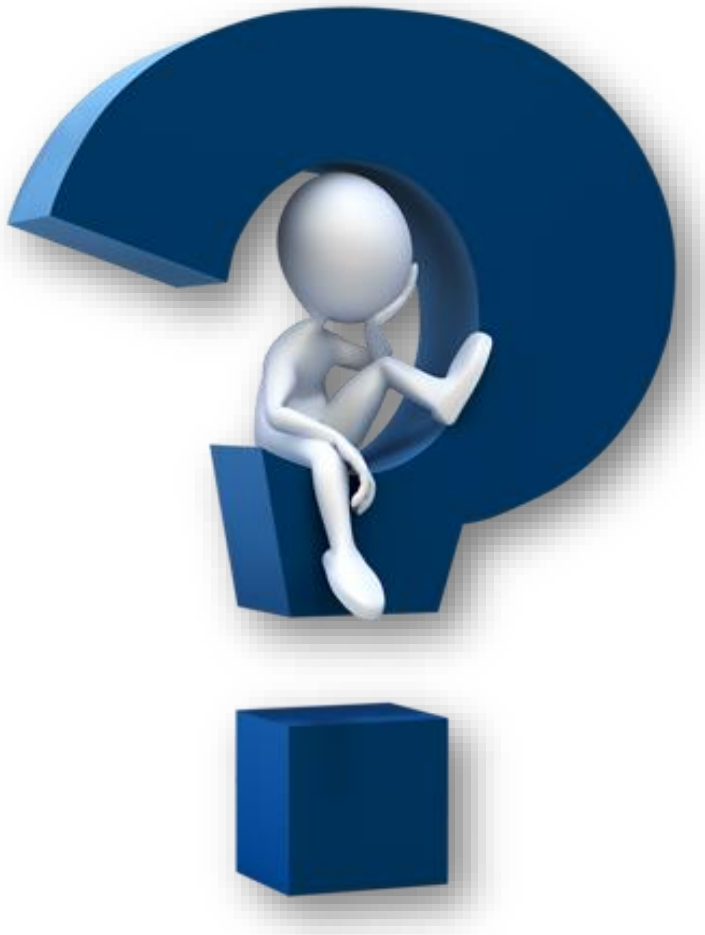

Supplement: Supplementary file 1 [file datasheet1.pdf]
