# Supplementary material for: Towards a Machine Vision-Based Yield Monitor for the Counting and Quality Mapping of Shallots
Source: Front Robot AI. 2021 Apr 16;8:627067. doi: 10.3389/frobt.2021.627067 (PMC8146908; doi:10.3389/frobt.2021.627067)
Supplement: Supplementary file 2 [file datasheet2.pdf]

Appendix A: Python Code

A-1 Initial Version of Python Code (Feasibility Study)

```
# -*- coding: utf-8 -*-
"""
Machine Vision Yield Monitor Program
@author: amanda
"""

import numpy as np
import cv2
import os
import math
import matplotlib.pyplot as plt

# Creates an elliptical structuring element for the opening/closing operations
# Elliptical Kernel

"""
>>> cv2.getStructuringElement(cv2.MORPH_ELLIPSE,(5,5))
array([[0, 0, 1, 0, 0],
       [1, 1, 1, 1, 1],
       [1, 1, 1, 1, 1],
       [1, 1, 1, 1, 1],
       [0, 0, 1, 0, 0]], dtype=uint8)
"""

ellipse_kernel = cv2.getStructuringElement(cv2.MORPH_ELLIPSE,(10,10))

# Define range of Red onion color in HSV
# onions
upper_red = np.array([46,180, 255])
lower_red = np.array([0,40,40])

def ellipse_perimeter(major_axis, minor_axis):
    a = major_axis/2
    b = minor_axis/2
    h = math.pow((a-b), 2)/math.pow((a+b), 2)
    perimeter = math.pi*(a+b)*(1+ 3*h/(10 + math.sqrt(4-3*h)))
    return perimeter

directory = 'C:/Users/amand/Desktop/Delfland Test 09152017/afternoon_test_09152017'

for root, dirs, filenames in os.walk(directory):
        for i, file in enumerate(filenames):
            imgpath = os.path.join(root,file) # Reconstructs the file path using the root_directory and current filename
            print(imgpath)

            #while(cap.isOpened()):
            #ret, frame = cap.read()
            # Resize Images
            img = cv2.imread(imgpath).copy()
            # Resize Images
```

```
            # Determines the new aspect ratio (r) and set the new dimensions for the im
age
            r = 500/img.shape[1]
            new_dim = (500, int(img.shape[0]*r))
            img = cv2.resize(img, new_dim, interpolation = cv2.INTER_AREA)
            # Convert the image to HSV colorspace
            blur = cv2.GaussianBlur(img,(7,7),0)
            img2 = cv2.cvtColor(blur, cv2.COLOR_BGR2HSV)
            # Threshold the HSV image to get only blue colors
            mask = cv2.inRange(img2, lower_red, upper_red)
            #mask = cv2.inRange(img2, lower_red, upper_red)
            opening = cv2.morphologyEx(mask, cv2.MORPH_OPEN, ellipse_kernel)
            closing = cv2.morphologyEx(opening, cv2.MORPH_CLOSE, ellipse_kernel)
            closing = cv2.GaussianBlur(closing,(3,3),0)
            # dilate makes the in range areas larger
            closing = cv2.dilate(closing, None, iterations=3)

            # Bitwise-AND mask and original image
            res = cv2.bitwise_and(img, img, mask = closing)
            dst, contours, hierarchy = cv2.findContours(closing, cv2.RETR_TREE, cv2.CHA
IN_APPROX_SIMPLE)

            for c in contours:
                moments = cv2.moments(c)
                if moments['m00'] != 0.0:
                    cx = int(moments['m10']/moments['m00'])
                    cy = int(moments['m01']/moments['m00'])
                    centroid = (cx,cy)
                    if len(c) > 5:
                        ellipse = cv2.fitEllipse(c)
                        major_axis,minor_axis = ellipse[1]
                        if (major_axis < 110) and (minor_axis<110):

                            print('major_axis: ')
                            print(major_axis)
                            print('minor_axis')
                            print(minor_axis)
                            perimeter = ellipse_perimeter(major_axis, minor_axis)
                            print('perimeter: ')
                            print(perimeter)
                            #if perimeter > 60: # Lobok
                            if (80 < perimeter) and (perimeter < 800): # Shallot onion

                                cv2.ellipse(img,ellipse,(0,0,255),2)
                    else:
                        pass


            cv2.imshow('Final Result', img)
            cv2.waitKey(1000)

cv2.destroyAllWindows()

      # End of program
```

A-2 Final Version of Python Code (Field Trial)

Yield Monitor Class

```
1.  # -*- coding: utf-8 -*-
2.
3.  ## USAGE cd to final_code
4.  # python YieldMonitor.py --conf conf.json
5.
6.  """
7.  Development of a Machine Vision Based Yield Monitor for Shallot Onions
8.  Precision Agriculture and Sensor Systems Group (PASS)
9.  McGill University, Department of Bioresource Engineering
10.
11. yield_monitor.py --- This is a yield monitoring program for the masters thesis of
12. Amanda Boatswain Jacques. This software detects onion shapes, classifies them by
13. size, and then exports them into a .CSV file with GPS data.
14. """
15.
16. # program Properties
17. __author__ = "Amanda Boatswain Jacques"
18. __version__ = 9.0
19.
20. # import the necessary python libraries
21. from datetime import datetime
22. import conf
23. import os
24. import pandas as pd
25. import serial
26. import sys
27. import time
28.
29. # computer vision
30. import cv2
31. import numpy as np
32. import preprocess_image
33. import size_calibration
34.
35. # create yield monitor class
36. class YieldMonitor:
37.     def __init__(self, config):
38.         # set current file path
39.         self.current_dir = sys.argv[0]
40.
41.         # load the configuration file
42.         if conf is None:
43.             raise ValueError
44.
45.         else:
46.             self.conf = conf.Conf(config)
47.             sources = self.conf["camera_sources"]
48.
49.         # initialize the camera
50.         for source in sources:
51.             try:
52.                 self.camera = cv2.VideoCapture(source)
53.
54.                 if self.camera.isOpened():
```

```
                    self.pretty_print("[INFO] CAMERA", "OK: Camera successfully opened"
)
                    self.pretty_print("[INFO] CAMERA", "Camera initialized!")

            except Exception as e:
                self.pretty_print("[ERROR] CAMERA", "Error: %s" % str(e))
                self.close()

        # create a directory for storing the images and .csv
        self.path = self.conf["external_drive"]
        date = datetime.strftime(datetime.now(), "%Y%m%d_%H%M" +"/")
        self.image_directory = self.path  + date
        self.result_directory = self.image_directory + "result_images/"
        self.pretty_print("[INFO] IMAGES", "Images will be saved in: " + self.image_dir
ectory)

        if not os.path.exists(self.image_directory):
            os.makedirs(self.image_directory)
            os.makedirs(self.result_directory)

    ### useful Functions
    def pretty_print(self, task, msg):
        # Pretty Print
        date = datetime.strftime(datetime.now(), '%d/%b/%Y %H:%M:%S')
        print('[%s] %s\t%s' % (date, task, msg))

   ### camera Functions
    def capture_image(self, write=False, ramp_frames = 40):
        """ Captures a single image from the camera and returns it in PNG format
        read is the easiest way to get a full image out of a VideoCapture object."""

        #self.pretty_print("[INFO] CAMERA", "Capturing photo...")

        # let the camera stabilize for 40 frames
        for i in range(ramp_frames):
            try:
                (retval, self.bgr) = self.camera.read()

            except Exception as e:
                self.pretty_print("[ERROR] CAMERA", "Error: %s" % str(e))
                self.close()

        if self.bgr is not None:
            cv2.imshow("Captured Image", self.bgr)
            cv2.waitKey(100)

            # save the image
                    if write == True:
                        date = datetime.strftime(datetime.now(), "%Y%m%d"+"_"+"%H%M%S")

                        #add directory here
                        self.filename = self.image_directory + date + ".png"
                        cv2.imwrite(self.filename, self.bgr)

                    else:
                        pass

                    return self.bgr

           # perform size calibration
           def calibrate_monitor(self):
```

```
113.                 self.pixel_metric = size_calibration.calibrate(self.conf["calibration_di
    rectory"])
114.                 self.pretty_print("[INFO] SIZE CALIBRATION", "Calibration completed.")
115.
116.                 return self.pixel_metric
117.
118.             # perform image processing and detect the onions in an image
119.             def find_onions(self, write=True):
120.                 original, preprocessed = preprocess_image.preprocess(self.bgr, resize=Fa
    lse)
121.                 self.small_count, self.medium_count, self.large_count, self.result = pre
    process_image.find_onion_contours(
122.                         preprocessed, original, self.pixel_metric)
123.
124.                 if write == True:
125.                     date = datetime.strftime(datetime.now(), "%Y%m%d"+"_"+"%H%M%S")
126.                     self.result_filename = self.result_directory + date + ".png"
127.                     cv2.imwrite(self.result_filename, self.result)
128.
129.
130.                 return(self.small_count, self.medium_count, self.large_count)
131.
132.             def init_gps(self):
133.                 """  Initialize the gps sensor and set the baudrate. """
134.                 COMNUMS = self.conf["gps_ports"]
135.                 self.gps = serial.Serial()
136.
137.                 self.pretty_print("[INFO] GPS", "Initializing GPS... ")
138.                 # detect the active gps port and save it
139.                 for port in COMNUMS:
140.                     try:
141.                          self.gps = serial.Serial(port, timeout = 0.2)
142.                          self.gps_port = port
143.                          # explicit close 'cause of delayed GC in java
144.                          #self.gps.close()
145.
146.                     except serial.SerialException:
147.                         pass
148.
149.                 if self.gps.isOpen():
150.                     # set the gps baudrate
151.                     self.gps.baudrate = self.conf["gps_baudrate"]
152.                     self.pretty_print("[INFO] GPS", "GPS at port %s with baud %s! " % (s
    elf.gps_port, self.gps.baudrate))
153.
154.                 else:
155.                     self.pretty_print("[ERROR] GPS", "GPS not found!.")
156.                     self.close()
157.
158.
159.             def get_position(self):
160.                 """ Get the current position (latitude, longitude, speed) of the image.
    """
161.                 # retrieve only the RMC sentences
162.                 code = "RMC"
163.
164.                 while True:
165.                     try:
166.                         line = self.gps.readline()
167.                         line = line.decode("utf-8")
168.                         #print(line)
```

```
169.
170.                         if line.find(code) > 0:
171.                             break
172.
173.                     except UnicodeDecodeError:
174.                         pass
175.
176.                 gps_data = line.split(",")
177.
178.                 # only report GPS sentences if an active valid fix was received
179.                 #if gps_data[2] == "V":
180.                 if gps_data[2] == "A":
181.                     self.latitude = gps_data[3]
182.                     self.latitude_char = gps_data[4]
183.                     self.longitude = gps_data[5]
184.                     self.longitude_char = gps_data[6]
185.                     self.speed = gps_data[7]
186.
187.                     if self.speed is not None:
188.                         self.speed = float(gps_data[7])*1.852
189.                         self.speed = format(self.speed, ".3f")
190.                 else:
191.                     pass
192.
193.                 return (self.latitude, self.longitude, self.speed)
194.
195.             def run(self):
196.                 """ Run the program continuously. Get captures,
197.                 analyze them, and then save the current position. """
198.
199.                 self.pretty_print("[INFO] RUNNING", "Running yield monitoring program. P
    ress CTRL+C to exit.")
200.
201.                 # open the GPS port, give some time for GPS and camera to stabilize
202.
203.                 time.sleep(5)
204.                 self.data = []
205.
206.                 while (True):
207.                     try:
208.                         self.capture_image(write =True)
209.                         small, medium, large = self.find_onions()
210.                         lat, long, speed = self.get_position()
211.                         self.log = [small, medium, large, lat, long, speed]
212.                         columns = ['S', 'M', 'L', 'Latitude', 'Longitude', 'Speed (km/h)
    ']
213.                         cv2.putText(self.result, str(columns), (10, 40), cv2.FONT_HERSHE
    Y_SIMPLEX, 0.8, (0,0,255), 2, cv2.LINE_AA)
214.                         cv2.putText(self.result, str(self.log), (10, 70), cv2.FONT_HERSH
    EY_SIMPLEX, 0.75, (255,255,255), 2, cv2.LINE_AA)
215.                         cv2.imshow("result", self.result)
216.                         cv2.waitKey(100)
217.
218.                         if self.filename is not None:
219.                             self.log = [small, medium, large, lat, long, speed, self.res
    ult_filename]
220.
221.                         self.data.append(self.log)
222.
223.                         print(self.log)
224.
```

```
                    except KeyboardInterrupt:
                        cv2.destroyAllWindows()
                        self.gps.close()
                        self.data = np.array(self.data)
                        if self.filename is not None:
                            self.df = pd.DataFrame(self.data, columns = ['Small Onions', 'Medium Onions', 'Large Onions', 'Latitude', 'Longitude', 'Speed', 'Filename'])
                        else:
                            self.df = pd.DataFrame(self.data, columns = ['Small Onions', 'Medium Onions', 'Large Onions', 'Latitude', 'Longitude', 'Speed'])
                        print(self.df)

                        break

                return self.df

            ### write everything to csv
            def save_log(self):
                time.sleep(2)
                test_filename = input("Please enter the name of the results file (use only numbers, letters and underscores):  " )
                self.pretty_print("[INFO] SAVING", "Saving results from test run as %s " % (test_filename + ".csv"))
                self.df.to_csv(self.conf["log_file_path"] + test_filename + ".csv")

            ### close the program
            def close(self):
                # shut down the program and delete camera source
                self.pretty_print("[INFO] WARN", "Shutdown triggered!")
                time.sleep(5)
                self.gps.close()
                self.camera.release()
                cv2.destroyAllWindows()

                sys.exit()

        # End of program
```

Image Preprocessing

```
# -*- coding: utf-8 -*-
"""
Created on Thu Apr 26 13:09:08 2018
preprocess_image_updated.py

@author: Amanda

Machine Vision Yield Monitor Program
"""

""" Import Libraries """
# import the necessary python libraries
import numpy as np
import cv2
import os
import math
from skimage.feature import peak_local_max
from skimage.morphology import watershed
from scipy import ndimage
```

```
import size_calibration

""" Define Functions and Variables """

pixel_metric = size_calibration.calibrate("./calibration_images_undistorted/")
print("The pixel metric is: ", pixel_metric)


def ellipse_perimeter(major_axis, minor_axis):
    a = major_axis/2
    b = minor_axis/2
    h = math.pow((a-b), 2)/math.pow((a+b), 2)
    perimeter = math.pi*(a+b)*(1+ 3*h/(10 + math.sqrt(4-3*h)))
    return perimeter

def auto_canny(image, sigma=0.60):
    # compute the median of the single channel pixel intensities
    v = np.median(image)
    # apply automatic Canny edge detection using the computed median
    lower = int(max(0, (1.0 - sigma) * v))
    upper = int(min(255, (1.0 + sigma) * v))
    edged = cv2.Canny(image, lower, upper)
    # return the edged image
    return edged

# creates an elliptical structuring element for the opening/closing operations
ellipse_kernel = cv2.getStructuringElement(cv2.MORPH_ELLIPSE, (12, 12))

# define range of Red onion color in HSV
Lupper_red = np.array([50, 255, 255])
Llower_red = np.array([0,40,0])

Uupper_red = np.array([180,255, 255])
Ulower_red = np.array([160,40,0])

lower_white = np.array([0, 0, 240], dtype = "uint8")
upper_white = np.array([60, 30, 255], dtype ="uint8")

image_directory = "C:/Users/Amanda/Documents/yield_monitor_results_copy/20180924_1411_u
ndistorted/"

# original preprocessing method
def preprocess_original(image):

    #cv2.imshow("Original Image", image)
    # perform Mean Shift Filtering
    shifted = cv2.pyrMeanShiftFiltering(image, 14, 50)

    # convert the image to HSV colorspace and blur
    blur = cv2.medianBlur(shifted, 9)
    blur = cv2.GaussianBlur(blur, (9,9),0)
    hsv = cv2.cvtColor(blur, cv2.COLOR_BGR2HSV)
    h, s, v = cv2.split(hsv)

    """
    ## perform mean subtraction normalization

    hue_mean = np.ones(h.shape, dtype=np.uint8)*np.mean(h)
    hue_mean = hue_mean.astype(np.uint8)
    subtracted_hue = cv2.subtract(h, hue_mean)

```

```
    sat_mean = np.ones(s.shape, dtype=np.uint8)*np.mean(s)
    sat_mean = sat_mean.astype(np.uint8)
    subtracted_sat = cv2.subtract(s, sat_mean)

    val_mean = np.ones(v.shape, dtype=np.uint8)*np.mean(v)
    val_mean = val_mean.astype(np.uint8)
    subtracted_val = cv2.subtract(v, val_mean)
    subtracted = cv2.merge((subtracted_hue, subtracted_sat, subtracted_val))

    cv2.imshow("Mean Substracted Image", subtracted)
    cv2.imshow("Mean Subtracted Hue Channel", subtracted_hue)
    cv2.imshow("Mean Subtracted Saturation Channel", subtracted_sat)
    cv2.imshow("Mean Subtracted Value Channel", subtracted_val)

    #subt_ret,subt_thresh = cv2.threshold(subtracted_hue,0,255,cv2.THRESH_BINARY+cv2.THRESH_OTSU)

    """
    # threshold the HSV image to get only red colors
    color_mask_lower = cv2.inRange(hsv, Llower_red, Lupper_red)
    color_mask_upper = cv2.inRange(hsv, Ulower_red, Uupper_red)
            color_mask_white = cv2.inRange(hsv, lower_white, upper_white)
            color_mask = color_mask_lower + color_mask_upper +color_mask_white
            color_opening = cv2.morphologyEx(color_mask, cv2.MORPH_OPEN, ellipse_kernel)

            color_closing = cv2.morphologyEx(color_opening, cv2.MORPH_CLOSE, ellipse_kernel)
            cv2.imshow("Color Segmentation", color_closing)

            ## chromacity calculations
            # split the BGR channels
            b, g, r = cv2.split(blur)
            b = b.astype('float')
            g = g.astype('float')
            r = r.astype('float')

            # add the 3 channels together
            merged = np.add(r, b)
            merged = np.add(merged, g)

            # calculate red intensity
            R_I = 3*r - b -g
            R_I = R_I.astype('uint8')
            cv2.imshow("Red Intensity Image", R_I)

            # calculate chromacity of the red channel
            chro_r = np.divide(r, merged)*255
            chro_r = chro_r.astype('uint8')
            cv2.imshow("Chromacity Image", chro_r)

            # equalize red chromacity and apply a median blur, remove noise
            equ = cv2.equalizeHist(chro_r)
            equ = cv2.medianBlur(equ, 9)
            grayscaled = R_I.copy()
            grayscaled = cv2.equalizeHist(grayscaled)
            grayscaled= cv2.GaussianBlur(grayscaled, (9,9),0)

            chro_edged = cv2.Canny(grayscaled, 180, 255)
            #chro_edged = cv2.dilate(chro_edged, ellipse_kernel, iterations=1)
            cv2.imshow("Chromacity Edged", chro_edged)
            final_mask = cv2.bitwise_and(chro_edged, color_closing)
```

```
138.             cv2.imshow("Final Mask", final_mask)
139.
140.             small, medium, large, result = find_onion_contours(final_mask.copy(),
141.                                                                image.copy(), pixel_metri
    c)
142.             cv2.imshow("Final Result", result)
143.             cv2.waitKey(2000)
144.             return (small, medium, large), result
145.
146.         def preprocess_watershed(image):
147.             # create a copy of the image to draw on
148.             clone = image.copy()
149.             #cv2.imshow("Original", clone)
150.             wts_result = np.zeros(image.shape, dtype=np.uint8)
151.             # perform Mean Shift Filtering
152.             #shifted = cv2.pyrMeanShiftFiltering(image, 14, 50)
153.             #cv2.imshow("Mean Shift Filtering", shifted)
154.             shifted = clone.copy()
155.             # convert the image to HSV colorspace and blur
156.             blur = cv2.medianBlur(shifted, 9)
157.             blur = cv2.GaussianBlur(blur, (9,9),0)
158.             cv2.imshow("Blurred Image", blur)
159.             hsv = cv2.cvtColor(blur, cv2.COLOR_BGR2HSV)
160.             h, s, v = cv2.split(hsv)
161.
162.             # threshold the HSV image to get only red colors
163.             color_mask_lower = cv2.inRange(hsv, Llower_red, Lupper_red)
164.             color_mask_upper = cv2.inRange(hsv, Ulower_red, Uupper_red)
165.             color_mask_white = cv2.inRange(hsv, lower_white, upper_white)
166.             color_mask = color_mask_lower + color_mask_upper + color_mask_white
167.             cv2.imshow("Color Mask", color_mask)
168.             color_opening = cv2.morphologyEx(color_mask, cv2.MORPH_OPEN, ellipse_kernel)

169.             cv2.imshow("Opening", color_opening)
170.             color_closing = cv2.morphologyEx(color_opening, cv2.MORPH_CLOSE, ellipse_ker
    nel)
171.             cv2.imshow("Closing", color_closing)
172.
173.             # sure background area
174.             sure_bg = color_closing.copy()
175.
176.             # Finding sure foreground area
177.             dist_transform =  ndimage.distance_transform_edt(sure_bg)
178.             cv2.imshow("Distance Transform CV2", dist_transform/np.max(dist_transform[:,
    :]))
179.             thresh = color_closing.copy()
180.
181.             # compute the exact Euclidean distance from every binary
182.             # pixel to the nearest zero pixel, then find peaks in this distance map
183.             localMax = peak_local_max(dist_transform, indices=False, min_distance=20,
184.                                       labels=thresh)
185.             # perform a connected component analysis on the local peaks,
186.             # using 8-connectivity, then appy the Watershed algorithm
187.             markers = ndimage.label(localMax, structure=np.ones((3, 3)))[0]
188.             labels = watershed(-dist_transform, markers, mask=thresh)
189.             print("[INFO] {} unique segments found".format(len(np.unique(labels)) - 1))

190.             # loop over the unique labels returned by the Watershed
191.             # algorithm
192.
193.             total_small = int()
```

```
194.            total_medium = int()
195.            total_large = int()
196.
197.            if (len(np.unique(labels)) - 1) >= 1:
198.                for label in np.unique(labels):
199.                    # if the label is zero, we are examining the 'background'
200.                    # so simply ignore it
201.                    if label == 0:
202.                        continue
203.                    # otherwise, allocate memory for the label region and draw
204.                    # it on the mask
205.                    mask = np.zeros(color_closing.shape, dtype="uint8")
206.                    mask[labels == label] = 255
207.                    #cv2.imshow("Watershed Mask", mask)
208.                    #cv2.waitKey(300)
209.                    small, medium, large, wts_result = find_onion_contours(mask, clone,
210.                                                                           pixel_metric)
211.
212.                    clone = wts_result.copy()
213.
214.                    total_small = total_small + small
215.                    total_medium = total_medium + medium
216.                    total_large = total_large + large
217.
218.            if not wts_result.any():
219.                wts_result = image.copy()
220.
221.            #cv2.imshow("Final Result", wts_result)
222.            #cv2.waitKey(500)
223.
224.            return (total_small, total_medium, total_large), wts_result
225.
226.        def find_onion_contours(mask, original, pixel_metric):
227.            # initialize lists of all the onion types present in the image
228.            small_onions = []
229.            medium_onions = []
230.            large_onions = []
231.            dst, contours, hierarchy = cv2.findContours(mask, cv2.RETR_LIST,
232.                                                        cv2.CHAIN_APPROX_SIMPLE)
233.            result = original.copy()
234.
235.            for c in contours:
236.
237.                moments = cv2.moments(c)
238.                if moments['m00'] != 0.0:
239.                    cx = int(moments['m10']/moments['m00'])
240.                    cy = int(moments['m01']/moments['m00'])
241.                    centroid = (int(cx), int(cy))
242.                    ((x, y), radius) = cv2.minEnclosingCircle(c)
243.
244.                    if len(c) > 5:
245.                        #print("radius : ", radius)
246.                        #if (radius<60) and (radius>16):
247.                        ellipse = cv2.fitEllipse(c)
248.                        major_axis, minor_axis = ellipse[1]
249.                        width = radius/pixel_metric
250.                        #print("width in mm", width)
251.
252.                        try:
```

```
                            aspect_ratio = minor_axis/major_axis
                            #print("aspect ratio :", aspect_ratio)

                        except ZeroDivisionError:
                            pass

                        # filter the onions by size
                        if 15 <=  width < 22: # small onion
                            color = (255, 0, 0) # Blue
                            small_onions.append(c)
                        if (width > 22) and (width < 27): # Medium Onion
                            medium_onions.append(c)
                            color = (0, 255, 0) # Green
                        if (width > 27) and (width < 40): # Large Onion
                            large_onions.append(c)
                            color = (0, 0, 255) # Red

                        if any((small_onions, medium_onions, large_onions)):
                            if  15 <= width <= 40:
                                # draw the centroid of the circle as well as the onion b
    order
                                cv2.circle(result, centroid, 3, (255, 255, 0), -1)
                                #cv2.ellipse(result, ellipse, color, 2)
                                cv2.circle(result, (int(x), int(y)), int(radius), color,
     2)
                else:
                    pass

            # return the counts for each onion category
            return len(small_onions), len(medium_onions), len(large_onions), result
```

Config File Loading

```
# import the necessary packages
from json_minify import json_minify
import json

class Conf:
    def __init__(self, confPath):
        # load and store the configuration and update the object's dictionary
        conf = json.loads(json_minify(open(confPath).read()))
        self.__dict__.update(conf)

    def __getitem__(self, k):
        # return the value associated with the supplied key
        return self.__dict__.get(k, None)

```

GPS Sentence Parsing

```
# -*- coding: utf-8 -*-
"""
@author: Amanda
"""
```

```
5.
6.  import serial
7.
8.  port ="COM7" # Add to config file
9.  BAUDRATE = 38400 # Add to config file
10.
11. gps = serial.Serial(port)
12. gps.baudrate = BAUDRATE
13.
14. while True:
15.     # flush the gps serial port
16.     #gps.flushInput()
17.     try:
18.         line = gps.readline()
19.         line = line.decode("utf-8")
20.         gps_data = line.split(",")
21.
22.         # retrieve only the RMC sentences
23.         if gps_data[0] == "$GPRMC":
24.             # only report GPS sentences if an active valid fix was received
25.             #if gps_data[2] == "A":
26.             if gps_data[2] == "V":
27.                 latitude = gps_data[3]
28.                 latitude_char = gps_data[4]
29.
30.                 longitude = gps_data[5]
31.                 longitude_char = gps_data[6]
32.                 speed = gps_data[7]
33.                 #speed = format(speed, ".3f")
34.                 #speed = str(speed)
35.
36.                 print('%s %s %s %s %s %s' % (latitude, latitude_char, longitude,
37.                       longitude_char, speed, "km/h" ))
38.
39.
40.                 print('%s %s %s %s ' % (latitude, latitude_char, longitude,
41.                       longitude_char))
42.
43.     except KeyboardInterrupt:
44.         gps.close()
45.         break
```

Main File

```
1.  # -*- coding: utf-8 -*-
2.  """
3.
4.  @author: Amanda
5.  """
6.  import YieldMonitor
7.  import time
8.  import argparse
9.
10. # construct the argument parse and parse the arguments
11.
12. ap = argparse.ArgumentParser()
13. ap.add_argument("-c", "--
    config", required=True, help="path to the configuration file")
14. args = vars(ap.parse_args())
```

```
15.
16. YM = YieldMonitor.YieldMonitor(args["config"])
17. time.sleep(2)
18.
19. YM.pretty_print("[INFO] RUNNING", "Yield Monitor initialized!")
20. YM.calibrate_monitor()
21. time.sleep(2)
22. YM.init_gps()
23.
24. YM.run()
25. YM.save_log()
26.
27.
28. YM.close()
```

Image Preprocessing (Updated post field trials)

```
1.  # -*- coding: utf-8 -*-
2.  """
3.  preprocess_image_updated.py
4.
5.  @author: Amanda
6.
7.  Machine Vision Yield Monitor Program
8.  """
9.
10. """ Import Libraries """
11. # import the necessary python libraries
12. import numpy as np
13. import cv2
14. import os
15. import math
16. from skimage.feature import peak_local_max
17. from skimage.morphology import watershed
18. from scipy import ndimage
19. import size_calibration
20.
21. """ Define Functions and Variables """
22.
23. pixel_metric = size_calibration.calibrate("./calibration_images_undistorted/")
24. print("The pixel metric is: ", pixel_metric)
25.
26.
27. def ellipse_perimeter(major_axis, minor_axis):
28.     a = major_axis/2
29.     b = minor_axis/2
30.     h = math.pow((a-b), 2)/math.pow((a+b), 2)
31.     perimeter = math.pi*(a+b)*(1+ 3*h/(10 + math.sqrt(4-3*h)))
32.     return perimeter
33.
34. def auto_canny(image, sigma=0.60):
35.     # compute the median of the single channel pixel intensities
36.     v = np.median(image)
37.     # apply automatic Canny edge detection using the computed median
38.     lower = int(max(0, (1.0 - sigma) * v))
39.     upper = int(min(255, (1.0 + sigma) * v))
40.     edged = cv2.Canny(image, lower, upper)
41.     # return the edged image
42.     return edged
```

```
43.
44. # creates an elliptical structuring element for the opening/closing operations
45. ellipse_kernel = cv2.getStructuringElement(cv2.MORPH_ELLIPSE, (12, 12))
46.
47. # define range of Red onion color in HSV
48. Lupper_red = np.array([50, 255, 255])
49. Llower_red = np.array([0,40,0])
50.
51. Uupper_red = np.array([180,255, 255])
52. Ulower_red = np.array([160,40,0])
53.
54.
55. lower_white = np.array([0, 0, 240], dtype = "uint8")
56. upper_white = np.array([60, 30, 255], dtype ="uint8")
57.
58. image_directory = "C:/Users/Amanda/Documents/yield_monitor_results_copy/20180924_1411_u
    ndistorted/"
59.
60. # original preprocessing method
61. def preprocess_original(image):
62.
63.     # perform Mean Shift Filtering
64.     shifted = cv2.pyrMeanShiftFiltering(image, 14, 50)
65.
66.     # convert the image to HSV colorspace and blur
67.     blur = cv2.medianBlur(shifted, 9)
68.     blur = cv2.GaussianBlur(blur, (9,9),0)
69.     hsv = cv2.cvtColor(blur, cv2.COLOR_BGR2HSV)
70.     h, s, v = cv2.split(hsv)
71.
72.     """
73.     ## perform mean subtraction normalization
74.
75.     hue_mean = np.ones(h.shape, dtype=np.uint8)*np.mean(h)
76.     hue_mean = hue_mean.astype(np.uint8)
77.     subtracted_hue = cv2.subtract(h, hue_mean)
78.
79.     sat_mean = np.ones(s.shape, dtype=np.uint8)*np.mean(s)
80.     sat_mean = sat_mean.astype(np.uint8)
81.     subtracted_sat = cv2.subtract(s, sat_mean)
82.
83.     val_mean = np.ones(v.shape, dtype=np.uint8)*np.mean(v)
84.     val_mean = val_mean.astype(np.uint8)
85.     subtracted_val = cv2.subtract(v, val_mean)
86.     subtracted = cv2.merge((subtracted_hue, subtracted_sat, subtracted_val))
87.
88.     cv2.imshow("Mean Substracted Image", subtracted)
89.     cv2.imshow("Mean Subtracted Hue Channel", subtracted_hue)
90.     cv2.imshow("Mean Subtracted Saturation Channel", subtracted_sat)
91.     cv2.imshow("Mean Subtracted Value Channel", subtracted_val)
92.
93.     #subt_ret,subt_thresh = cv2.threshold(subtracted_hue,0,255,cv2.THRESH_BINARY+cv2.TH
    RESH_OTSU)
94.
95.     """
96.     # threshold the HSV image to get only red colors
97.     color_mask_lower = cv2.inRange(hsv, Llower_red, Lupper_red)
98.     color_mask_upper = cv2.inRange(hsv, Ulower_red, Uupper_red)
99.     color_mask_white = cv2.inRange(hsv, lower_white, upper_white)
100.          color_mask = color_mask_lower + color_mask_upper +color_mask_white
```

```
            color_opening = cv2.morphologyEx(color_mask, cv2.MORPH_OPEN, ellipse_kernel)

            color_closing = cv2.morphologyEx(color_opening, cv2.MORPH_CLOSE, ellipse_ker
    nel)

            ## chromacity calculations
            # split the BGR channels
            b, g, r = cv2.split(blur)
            b = b.astype('float')
            g = g.astype('float')
            r = r.astype('float')

            # add the 3 channels together
            merged = np.add(r, b)
            merged = np.add(merged, g)

            # calculate red intensity
            R_I = 3*r - b -g
            R_I = R_I.astype('uint8')

            # calculate chromacity of the red channel
            chro_r = np.divide(r, merged)*255
            chro_r = chro_r.astype('uint8')

            # equalize red chromacity and apply a median blur, remove noise
            equ = cv2.equalizeHist(chro_r)
            equ = cv2.medianBlur(equ, 9)

            grayscaled = R_I.copy()
            grayscaled = cv2.equalizeHist(grayscaled)
            grayscaled= cv2.GaussianBlur(grayscaled, (9,9),0)

            chro_edged = cv2.Canny(grayscaled, 180, 255)
            chro_edged = cv2.dilate(chro_edged, ellipse_kernel, iterations=1)
            final_mask = cv2.bitwise_and(chro_edged, color_closing)
            small, medium, large, result = find_onion_contours(final_mask.copy(),
                                                               image.copy(), pixel_metri
    c)

            return (small, medium, large), result

        def preprocess_watershed(image):
            # create a copy of the image to draw on
            clone = image.copy()
            wts_result = np.zeros(image.shape, dtype=np.uint8)
            # perform Mean Shift Filtering
            shifted = cv2.pyrMeanShiftFiltering(image, 14, 50)

            # convert the image to HSV colorspace and blur
            blur = cv2.medianBlur(shifted, 9)
            blur = cv2.GaussianBlur(blur, (9,9),0)
            hsv = cv2.cvtColor(blur, cv2.COLOR_BGR2HSV)
            h, s, v = cv2.split(hsv)

            # threshold the HSV image to get only red colors
            color_mask_lower = cv2.inRange(hsv, Llower_red, Lupper_red)
            color_mask_upper = cv2.inRange(hsv, Ulower_red, Uupper_red)
            color_mask_white = cv2.inRange(hsv, lower_white, upper_white)
            color_mask = color_mask_lower + color_mask_upper + color_mask_white
            color_opening = cv2.morphologyEx(color_mask, cv2.MORPH_OPEN, ellipse_kernel)
```

```
158.            color_closing = cv2.morphologyEx(color_opening, cv2.MORPH_CLOSE, ellipse_ker
    nel)
159.
160.            # sure background area
161.            sure_bg = color_closing.copy()
162.
163.            # Finding sure foreground area
164.            dist_transform =  ndimage.distance_transform_edt(sure_bg)
165.            #cv2.imshow("Distance Transform CV2", dist_transform/np.max(dist_transform[:
    ,:]))
166.            thresh = color_closing.copy()
167.
168.            # compute the exact Euclidean distance from every binary
169.            # pixel to the nearest zero pixel, then find peaks in this distance map
170.            localMax = peak_local_max(dist_transform, indices=False, min_distance=20,
171.                                      labels=thresh)
172.            # perform a connected component analysis on the local peaks,
173.            # using 8-connectivity, then appy the Watershed algorithm
174.            markers = ndimage.label(localMax, structure=np.ones((3, 3)))[0]
175.            labels = watershed(-dist_transform, markers, mask=thresh)
176.            print("[INFO] {} unique segments found".format(len(np.unique(labels)) - 1))

177.
178.            # loop over the unique labels returned by the Watershed
179.            # algorithm
180.
181.            total_small = int()
182.            total_medium = int()
183.            total_large = int()
184.
185.            if (len(np.unique(labels)) - 1) >= 1:
186.                for label in np.unique(labels):
187.                    # if the label is zero, we are examining the 'background'
188.                    # so simply ignore it
189.                    if label == 0:
190.                        continue
191.                    # otherwise, allocate memory for the label region and draw
192.                    # it on the mask
193.                    mask = np.zeros(color_closing.shape, dtype="uint8")
194.                    mask[labels == label] = 255
195.                    small, medium, large, wts_result = find_onion_contours(mask, clone,

196.                                                                           pixel_metric)

197.
198.                    clone = wts_result.copy()
199.
200.                    total_small = total_small + small
201.                    total_medium = total_medium + medium
202.                    total_large = total_large + large
203.
204.            if not wts_result.any():
205.                wts_result = image.copy()
206.
207.            return (total_small, total_medium, total_large), wts_result
208.
209.        def find_onion_contours(mask, original, pixel_metric):
210.            # initialize lists of all the onion types present in the image
211.            small_onions = []
212.            medium_onions = []
213.            large_onions = []
```

```
214.            dst, contours, hierarchy = cv2.findContours(mask, cv2.RETR_LIST,
215.                                                        cv2.CHAIN_APPROX_SIMPLE)
216.            result = original.copy()
217.
218.            for c in contours:
219.
220.                moments = cv2.moments(c)
221.                if moments['m00'] != 0.0:
222.                    cx = int(moments['m10']/moments['m00'])
223.                    cy = int(moments['m01']/moments['m00'])
224.                    centroid = (int(cx), int(cy))
225.                    ((x, y), radius) = cv2.minEnclosingCircle(c)
226.
227.                    if len(c) > 5:
228.                        #print("radius : ", radius)
229.                        #if (radius<60) and (radius>16):
230.                        ellipse = cv2.fitEllipse(c)
231.                        major_axis, minor_axis = ellipse[1]
232.                        width = radius/pixel_metric
233.                        #print("width in mm", width)
234.
235.                        try:
236.                            aspect_ratio = minor_axis/major_axis
237.                            #print("aspect ratio :", aspect_ratio)
238.
239.                        except ZeroDivisionError:
240.                            pass
241.
242.                        # filter the onions by size
243.                        if 15 <=  width < 25: # small onion
244.                            color = (255, 0, 0) # Blue
245.                            small_onions.append(c)
246.                        if (width > 25) and (width < 45): # Medium Onion
247.                            medium_onions.append(c)
248.                            color = (0, 255, 0) # Green
249.                        if (width > 45) and (width < 50): # Large Onion
250.                            large_onions.append(c)
251.                            color = (0, 0, 255) # Red
252.
253.                        if any((small_onions, medium_onions, large_onions)):
254.                            if  15 <= width <= 65:
255.                                # draw the centroid of the circle as well as the onion b
    order
256.                                cv2.circle(result, centroid, 3, (255, 255, 0), -1)
257.                                #cv2.ellipse(result, ellipse, color, 2)
258.                                cv2.circle(result, (int(x), int(y)), int(radius), color,
     2)
259.                else:
260.                    pass
261.
262.            # return the counts for each onion category
263.            return len(small_onions), len(medium_onions), len(large_onions), result
```

Statistical Analysis

```
# -*- coding: utf-8 -*-
"""
Created on Mon Feb 8 14:51:24 2019

@author: Amanda
"""

import numpy as np
import matplotlib.pyplot as plt
import pandas as pd
from scipy import stats

file_path = "C:/Users/Amanda/Documents/Thesis/Statistics/ks_data.xls"
print(file_path)

data = pd.read_excel(file_path, sheet_name="large_class", names = ["Predicted Diameter (mm)","True Diameter (mm)"])

# create plot area
fig, ax = plt.subplots(figsize=(8, 4))

n_bins = 50
predicted = np.asarray(data["Predicted Diameter (mm)"])
true = np.asarray(data["True Diameter (mm)"])


n, bins, patches = ax.hist(predicted, n_bins, density=True, histtype='step',
                           cumulative=True, label='Predicted Diameter')

n, bins, patches = ax.hist(true, n_bins, density=True, histtype='step',
                           cumulative=True, label='True Diameter')


# Compute the Kolmogorov-Smirnov statistic on the 2 gathered samples.
rs_statistic, p_value = stats.ks_2samp(predicted, true)

print("RS Statistic :", rs_statistic)
print("P Value:", p_value)


# tidy up the figure
ax.grid(True)
ax.set_title('Large Class: KS-
Test Comparison Cumulative Fraction Plot', fontname="Arial", fontsize = "large")
ax.legend(loc='right', prop={'size': 10})
ax.set_xlabel('Diameter (mm)', fontname="Arial", fontsize = "large")
ax.set_ylabel('Cumulative Fraction', fontname="Arial", fontsize = "large")

plt.show()
```

Appendix B: Hardware Specifics

B-1 Prototype Camera

Table B-1. Prototype Camera specifications

**Attribute**	**Value**
Brand Name	ELP
Resolution	2.0 Megapixel 1080P
Sensor	1/2.7" CMOS
Picture Format	MJPEG or YUY2 optional
USB	Protocal USB2.0 HS/FS
Exposure	Auto exposure AEC Support
White Balance	Auto white blance AEB Support
Effective pixels	1920 (H) x 1080 (V) pixels<br>1280 (H) x 1024 (V) pixels<br>1280 (H) x 720 (V) pixels<br>1024 (H) x 768 (V) pixels<br>800 (H) x 600 (V) pixels<br>640 (H) x 480 (V) pixels<br>352(H) x 288 (V) pixels<br>320 (H) x 240 (V) pixels
Performance	1920 (H) x 1080 (V) pixels MJPEG 30fps YUY2 6fps<br>1280 (H) x 1024 (V) pixels MJPEG 30fps YUY2 6fps<br>1280 (H) x 720 (V) pixels MJPEG 60fps YUY2 9fps<br>1024 (H) x 768 (V) pixels MJPEG 30fps YUY2 9fps<br>800 (H) x 600 (V) pixels MJPEG 60fps YUY2 21fps<br>640 (H) x 480 (V) pixels MJPEG 120fps YUY2 30fps
Voltage	DC 5-V/current 150mA
Size	Size 32x32mm/38*38
Work Temperature	DEGREES (-20~70)
Hardware Platform	PC; Mac; Android OS
Other Specifications	Adjustable parameters Brightness/Contrast/Color saturation /Definition/Gamma/WB<br>Night vision optional, Support IR Cut and IR board for night vision

B-2 Solid State Drive (SSD)

Table B-2. Solid state drive specifications

Attribute	Value
Brand Name	Samsung
Series	T5
Color	blue
Item Height	7.6 centimeter
Item Width	10 millimeters
Hard Disk Size	250 GB
Hard Disk Technology	Portable
Hardware Platform	PC;Mac;Android OS

B-3 GPS Sensor

Table B-3. GPS Sensor specifications

Attribute	Value
Brand Name	Garmin
Version	GPS 19x HVS (NMEA 0183)
Dimensions (DxH)	3 19/32" x 1 15/16" (91.6 mm x 49.5 mm)
Weight	7.1 oz (201 g)
Cable length	30 ft (9.14 m)
Temperature Range	-22° to 176° F (-30° to 80° C)
Compass-safe distance	5.9" (150 mm)
Power source input	8-33 Vdc, unregulated
Input current	40 mA at 12 Vdc

Appendix C: Design Drawings

C-1 Bracket Design

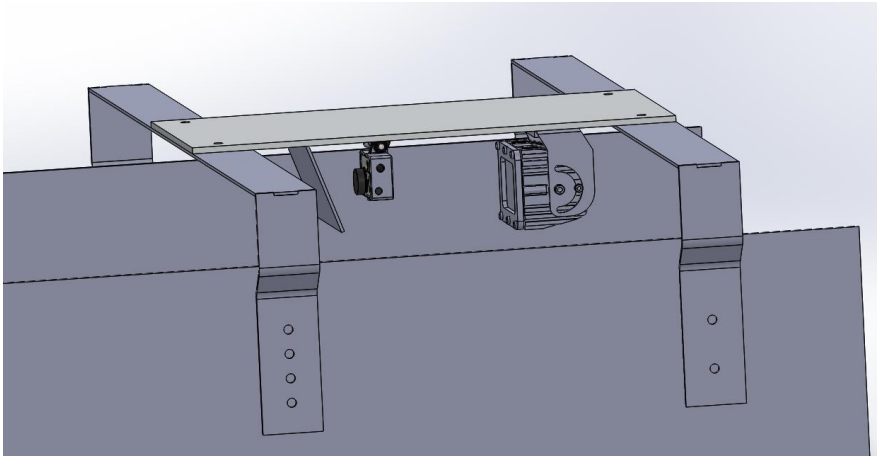

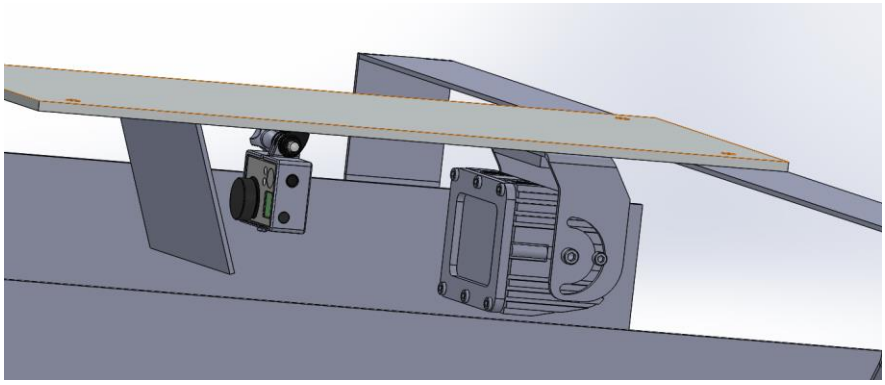

Appendix D: Additional Figures

D-1 Initial Size Calibration

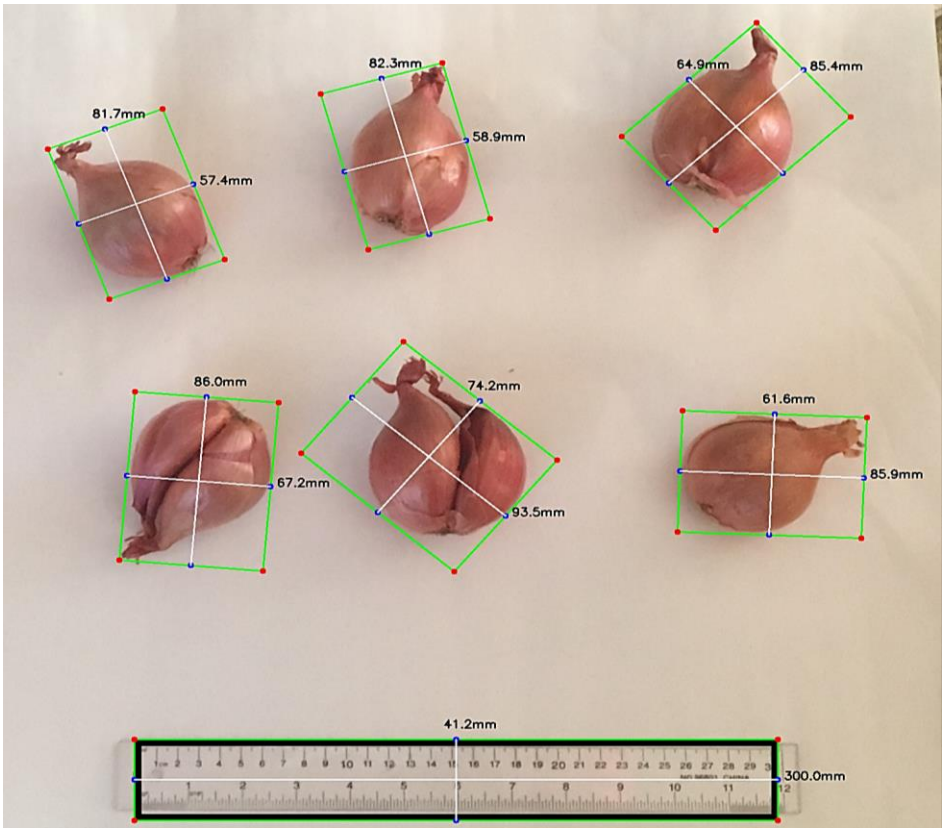

Figure D-1. Example image of the size calibration setup (section 3.2.7).

D-2 Modification of Size Classes

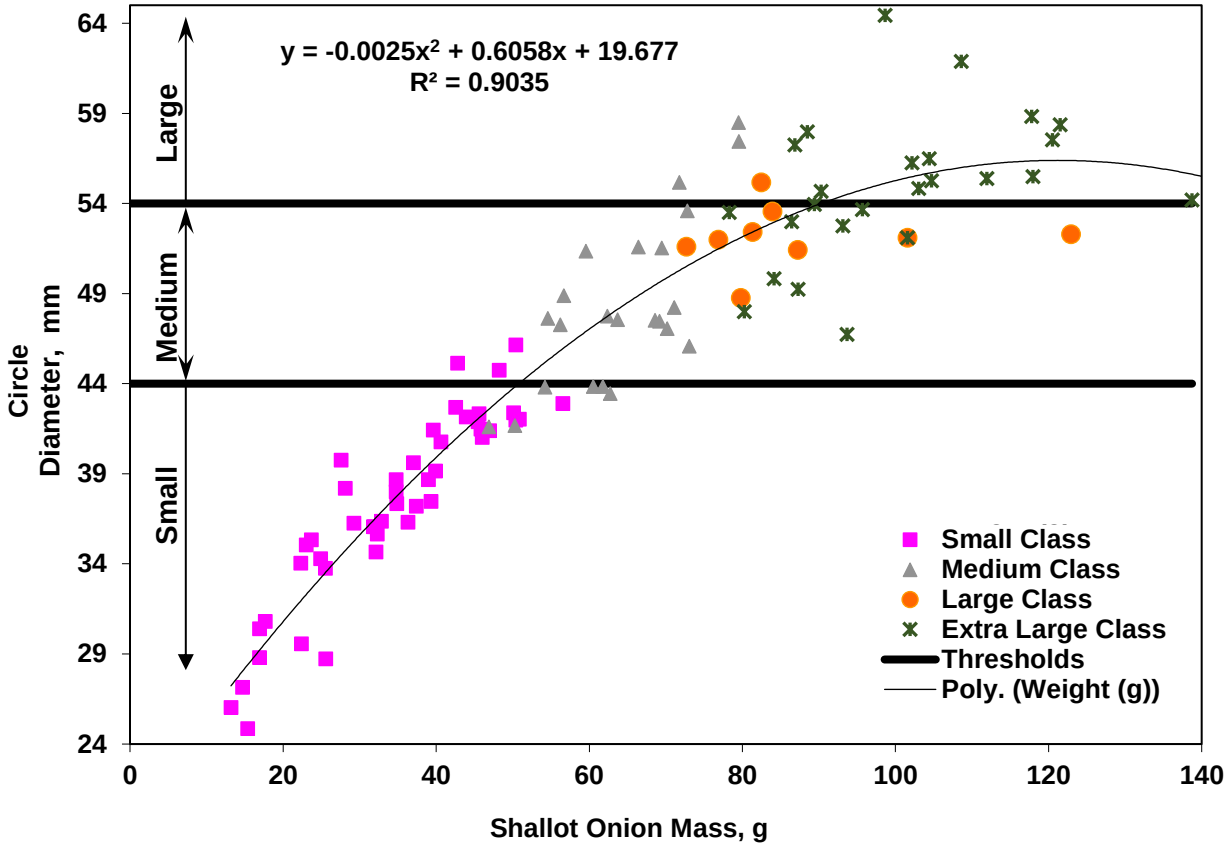

Figure D-2. Modification of the size classes for shallot onion classification.

Appendix E: Definition of Performance Metrics

Accuracy is the most important metric for evaluating the performance of a classification algorithm as it represents the algorithm's capacity to correctly classify the cases. It is calculated by summing the number of true positive and true negative classifications and dividing by the total number of classifications, and like all metrics is often multiplied by 100 to yield a percentage. Though it is the most important metric, accuracy alone is not enough to determine the strength of the algorithm and if it has learned the task at hand.

$$Accuracy = \frac{\Sigma\,\mathrm{TP} + \Sigma\,\mathrm{TN}}{\Sigma\,\mathrm{TP} + \Sigma\,\mathrm{FP} + \Sigma\,\mathrm{TN} + \Sigma\,\mathrm{FN}}$$

Precision also referred to as positive predictive value is used to determine the algorithm's capacity to correctly identify positive cases with respect to all the cases the algorithm has classified as positive or simply put it is a measure of the classifiers' exactness. It is calculated by dividing the number of true positives by the number of predicted condition positive which itself is a sum of the true positives and false positives.

$$Precision = \frac{\Sigma\,\mathrm{TP}}{\Sigma\,\mathrm{TP} + \Sigma\,\mathrm{FP}}$$
